# Global distribution and biogeography of ericoid mycorrhizal fungi

**DOI:** 10.1111/nph.71334

**Published:** 2026-06-12

**Authors:** Iñaki Odriozola, Tomáš Větrovský, Florian Barbi, Antonín Machac, Priscila Thiago Dobbler, Cristina Turcu, Michael E. Van Nuland, Clara Qin, Toby Kiers, Nadejda A. Soudzilovskaia, Petr Baldrian, Petr Kohout

**Affiliations:** ^1^ Institute of Microbiology of the Czech Academy of Sciences Vídeňská 1083 Prague 142 20 Czechia; ^2^ Society for the Protection of Underground Networks (SPUN) 3500 South DuPont Highway Suite EI‐101 Dover DE 19901 USA; ^3^ Faculty of Science Charles University Albertov 6 Prague 128 00 Czechia; ^4^ Amsterdam Institute for Life and Environment (A‐LIFE), Section Ecology & Evolution Vrije Universiteit Amsterdam Van der Boechorststraat 3 Amsterdam 1081 BT the Netherlands; ^5^ Centre for Environmental Sciences Hasselt University Martelarenlaan 42 Hasselt 3500 Belgium

**Keywords:** Ericaceae, fungal ecology, GlobalFungi, Helotiales, mycorrhiza, Sebacinales, species distribution modeling

## Abstract

- Ericoid mycorrhizal (ErM) fungi play a crucial role across terrestrial ecosystems, forming mutualistic symbiosis with Ericaceae and contributing to soil organic matter dynamics. However, compared to other fungal groups, their biogeography remains unknown.
- Here, we combined several analytical approaches to analyze a newly compiled, large‐scale dataset comprising 39 163 soil samples and more than 13 million ITS rRNA sequences assigned to ErM fungi. Specifically, we asked: What are the global patterns of ErM fungal species richness and relative abundance (out of all fungi) and their predictors, and how is the distribution of ErM fungi associated with soil carbon content at the global scale?
- We show that ErM fungi reach their highest species richness in very high latitudes. Soil chemistry is a stronger predictor of ErM fungal species richness than climate or ericoid vegetation cover. The relative abundance of ErM fungi is highest in soils with high surface carbon content, supporting their proposed role in soil carbon storage. Furthermore, we predict that climate change will reduce ErM fungal abundance across 38% of the land cover of their current global distribution.
- Our study shows distinct biogeographic patterns of ErM fungi compared with arbuscular and ectomycorrhizal fungi and indicates the vulnerability of ErM fungi to climate change.

## Graphical abstract

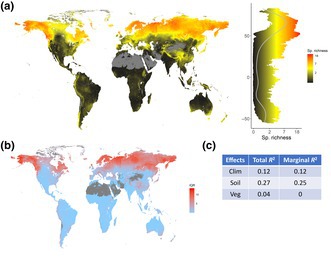

## Introduction

Compared to other major groups of organisms, the biogeography of mycorrhizal fungi remains largely enigmatic. These fungi form mutualistic symbiosis with > 80% of plant species (Brundrett & Tedersoo, 2018), and drive essential ecosystem functions and services, such as plant nutrition, pest control and soil aggregation (Martin & van der Heijden, 2024). Mycorrhizal fungi also play a fundamental role in nutrient cycling, with an estimated 3.6 billion tons of carbon allocated by host plants to their mycorrhizal symbionts annually (Hawkins *et al*., 2023). Among mycorrhizal types, arbuscular mycorrhiza (AM), ectomycorrhiza (EcM) and ericoid mycorrhiza (ErM) are geographically the most widespread, occurring at *c*. 96, 72 and 58% of global land area, respectively (Soudzilovskaia *et al*., 2019).

While ErM is globally widespread and appears highly important for carbon storage and turnover in soil, these organisms are less researched (Clemmensen *et al*., 2013, 2015; Ward *et al*., 2022). Past results for AM and EcM fungi might not extend to ErM fungi, given that their life history is different in several fundamental respects. Unlike obligatorily symbiotic AM and EcM fungi, which are unable to survive without their host plants, ErM fungi possess a wide range of extracellular enzymes that decompose complex organic compounds (Perotto *et al*., 2018), enabling them to live both symbiotically and as free‐living saprotrophs. Previous comparative genomic analyses indicated that the enzymatic repertoire of ErM fungi is much richer than that of EcM and AM, being rather similar to free‐living brown‐rot saprotrophs (Martino *et al*., 2018). In contrast to AM and EcM fungi, ErM fungi do not seem to depend on the presence of compatible host plants in the environment. For instance, ErM fungi commonly colonize a great diversity of non‐ErM host plants as endophytes without forming any specialized plant‐fungal interface for exchanging nutrients (Chambers *et al*., 2008; Vohník *et al*., 2013; Lukešová *et al*., 2015). But unlike free‐living saprotrophs, they can benefit from mycorrhizal or endophytic interactions with plants. Recent advances suggest potential differences in host dependence among ErM fungal lineages, with ErM fungi from the Sebacinales being more strongly host‐dependent, whereas helotialean ErM species may exhibit a more versatile ecology (Mielke *et al*., 2025). For these reasons, the biogeography of ErM cannot be easily gauged from previous studies on other mycorrhizal fungi, and their distributions remain largely unknown despite their key importance (Martin & van der Heijden, 2024).

Biogeography of fungi is a growing avenue of research. Facilitated by the onset of new methods, such as high throughput sequencing, previous findings have uncovered divergent and sometimes even contradictory patterns (Tedersoo *et al*., 2014, 2022; Větrovský *et al*., 2019; Abrego *et al*., 2024; Barbi *et al*., 2025). For example, total fungal species richness has been reported to follow not only the classical negative latitudinal diversity gradient (Tedersoo *et al*., 2014) but also its inverse form (Větrovský *et al*., 2019). Other work has shown that while AM fungal diversity seems to peak in the tropics, EcM fungi peak at temperate latitudes (Steidinger *et al*., 2019; Van Nuland *et al*., 2025). By contrast, ErM fungi remain comparatively understudied, but their diversity has been hypothesized to reach its maximum at very high latitudes (Read, 1991; Read & Perez‐Moreno, 2003; Kohout, 2017). This hypothesis, however, has yet to be systematically tested and confirmed using comprehensive data with global coverage. Consequently, we currently have only limited knowledge of the global diversity of ErM fungi and their main drivers (e.g. soil chemistry, climate and vegetation). Furthermore, we lack rigorous models of the geographic distributions of the individual ErM species and reliable estimates of their relative abundances in fungal communities. In fact, some of the ErM species rank among the most commonly identified species in fungal metabarcoding studies on a global scale (Větrovský *et al*., 2020). These knowledge gaps hinder our ability to properly evaluate the contribution of ErM fungi to global carbon dynamics and predict their responses to environmental change.

The number of described ErM fungal species is relatively small compared to other fungal guilds (Põlme *et al*., 2020), but these fungi are thought to be disproportionately abundant within a wide variety of ecosystems. Certain terrestrial ecosystems have a high abundance of ErM host plants (Bueno *et al*., 2017; Soudzilovskaia *et al*., 2019), including tundra habitats that are facing the most severe climate warming effects (Rantanen *et al*., 2022). The high share of ErM fungi in boreal forest soils has been identified as one of the main driving forces behind sequestration of soil carbon at high latitudes (Clemmensen *et al*., 2013). This is particularly driven by a recalcitrant ErM fungal necromass, due to the accumulation of melanin compounds in cell walls of many ErM fungal species (Fernandez *et al*., 2019). As a result, the relative abundance of ErM fungal taxa within soil fungal communities could provide a good proxy for their importance in local soil carbon storage and turnover, but this has not been examined at a global scale.

To address these challenges, we compiled ErM fungal sequence data to create high‐resolution global maps of ErM fungal species richness, estimate the relative abundance of ErM fungi world‐wide, and build the first species distribution models for individual ErM fungal species. Our study leverages the GlobalFungi database, the largest dataset of fungal internal transcript spacer (ITS) rRNA amplicon sequences assembled to date, encompassing 84 972 samples and containing more than 4.5 billion sequences. The unique size of this dataset allowed us to extract a comprehensive dataset of ErM fungi with global coverage. In this study, we use 39 163 soil samples containing 13 million ITS rRNA sequences belonging to ErM fungi. We examine the dataset, using Bayesian hierarchical models, joint species distribution modeling and geographic information systems (GIS). Our aims are fourfold: (1) reconstruct the global pattern of ErM fungal species diversity and relative abundance (out of total fungi) in soil fungal communities; (2) identify the best predictors of global ErM fungal species distributions, diversity, relative abundance and community composition; (3) assess whether ErM richness and relative abundance positively associate with soil organic carbon content at the global scale; (4) forecast ErM fungal species distributions, diversity and abundance shifts under global change. Consequently, our findings advance the knowledge of one of the most understudied and ecologically significant mycorrhizal symbiont groups on Earth.

## Materials and Methods

### Dataset characteristics

#### Selection of ericoid mycorrhizal fungi for the analysis

In this study, fungal taxa are represented by molecular taxa, so called Species Hypotheses (SH; Kõljalg *et al*., 2013), identified based on ITS rRNA sequences that are created with the aim of representing fungal species. SH possessing the ErM lifestyle were identified using the FungalTraits database (Põlme *et al*., 2020), as follows. We considered all SH belonging to the dynamic SH dataset (v.9.0; Abarenkov *et al*., 2024) to be ErM if they contained fungal ITS sequences from pure cultures, whose ErM lifestyle was supported by resynthesis experiments with ericoid host plants. Using this criterion, we identified 82 SHs.

However, this approach did not capture ErM fungi from the family Serendipitaceae. To address this limitation, we classified all SH belonging to the genus Serendipita as ericoid mycorrhizal (ErM) when they were reported from ericoid plant roots. This search resulted in 15 putative ErM SHs from the genus Serendipita.

In addition, several ITS sequences derived from fungal isolates with experimentally supported ErM lifestyles were not assigned to any existing SH. These sequences (*n* = 10) were downloaded from NCBI and used as reference sequences to define new SH.

By combining all three criteria described above, we identified a total of 107 ErM fungal taxa. A detailed overview of these ErM fungi is provided in Supporting Information Table S1. Although the strength of evidence for the ErM lifestyle varies among the 107 fungal taxa, we treat all of them as ErM for the purposes of this study. Hereafter, we refer to the identified ErM fungal SH as species.

#### Ericoid mycorrhizal lifestyle in fungi

It should be noted that, in this study, ErM fungi were defined as fungal taxa that have previously been shown to form hyphal coils in the root cells of ericoid plants in resynthesis experiments. This list is clearly incomplete, and future research will likely expand the number of recognized ErM fungal species. For example, Baba & Hirose (2024) reported evidence for a potential ErM lifestyle in some Archaeorhizomycetes taxa, suggesting that ErM fungi may also occur in previously unrecognized lineages. In addition, some ErM fungi may remain undetected because they are difficult to cultivate. This limitation likely explains why Sebacinales were experimentally shown to form ErM symbioses much later than the well‐known ErM taxa within Helotiales (Vohník *et al*., 2016).

#### Distribution data of ericoid mycorrhizal fungi

The occurrence and relative abundance data of the selected ErM fungal species were derived from the GlobalFungi database (https://globalfungi.com; Větrovský *et al*., 2020, 2023), Release 5. Together, the GlobalFungi database is an open‐access initiative that compiles the most comprehensive atlas of fungal species distribution based on molecular metabarcoding of environmental samples. The GlobalFungi database, Release 5 covers more than 80 000 samples. In the GlobalFungi database, the quality‐filtered individual ITS sequences are assigned to representative sequences of SH (v.9.0; Abarenkov *et al*., 2024) on a 98.5% similarity threshold (for more details, see Větrovský *et al*., 2020). Although the GlobalFungi database includes data originating from various substrates (e.g. air, dust, leaves, soil and water), we limited our analysis to soil samples only, which represents the largest pool of samples in the GlobalFungi database (39 163 samples in total). For each sample, occurrence of a specific SH in sample was defined as the presence of a sequence classified to that specific SH; species richness of ErM fungi was calculated as the number of ErM fungal SH present in a given sample, and, relative abundance of ErM fungi was calculated as the share of ITS sequences classified to ErM fungal SH divided by the total number of all fungal ITS sequences.

#### Sample metadata

All samples were assigned to continents, and all sites were categorized into biomes following the classification of Environment Ontology (http://www.ontobee.org/ontology/ENVO) to a maximum achievable depth for each sample. Climatic variables were retrieved for each sample based on its geographical location from the global CHELSA database. We considered the 19 bioclimatic variables derived from the monthly temperature and precipitation data, which are biologically meaningful variables routinely used in species distribution modeling (Karger *et al*., 2017). These variables represent different aspects of the climate, like long‐term averages, seasonality and climatic extremes, representing the period encompassing 1981–2010. We also used the same set of predicted bioclimatic variables for the 2041–2070 period under the ssp370 climate change scenario: this scenario assumes the climate projections generated by GCM climate models under the combined assumptions of the SSP3 socioeconomic pathway and the RCP7 greenhouse gas concentration trajectory. Soil characteristics were retrieved for each sample, based on its geographic location, from the global SoilGrids database (Hengl *et al*., 2017). We used soil pH, soil organic carbon concentration (SOC; dg kg^−1^) and soil nitrogen content (N; cg kg^−1^) from 0 to 5 cm of soil depth. Besides the climatic and soil characteristics, we also retrieved the information about the relative abundance of ErM plants (i.e. percentage of ErM vegetation cover out of total vegetation) in the local vegetation cover from Soudzilovskaia *et al*. (2019).

### Statistical analysis

After filtering out samples from the continent Antarctica, as well as mangroves, wetlands, flood grasslands, and anthropogenic and aquatic biomes, we retained 39 163 samples belonging to 14 018 locations with unique spatial coordinates (Table S2). Most of the samples were from Asia (14 283 samples and 2849 locations). However, Europe contributed most of the locations with unique spatial coordinates (12 810 samples and 5901 locations). They were followed in numbers of samples and locations by North America (6992 samples and 2248 locations), Australia (2430 samples and 1215 locations), Africa (1486 samples and 1207 locations) and South America (1162 samples and 598 locations). Due to uneven sampling intensity across continents, species accumulation curves showed that species diversity was more comprehensively captured for continents in the Northern Hemisphere. Nevertheless, when comparing species richness under similar sampling intensities, it was evident that Asia, Europe and North America harbor the highest diversity of ErM fungi, followed by South America and Australia with intermediate levels, and Africa with the lowest diversity (Fig. S1). To address spatial bias in sampling intensity, the modeling approach included nested random effects: Sampling points were nested within locations, which were in turn nested within clusters of locations and continents (see subsequently for details). This hierarchical structure ensured that areas with high sampling density were treated as spatially dependent observations rather than as independent samples from similar environmental conditions.

All models were fitted to randomly selected 80% of the 14 018 locations, which also represented *c*. 80% of the samples (hereafter the *training set*: containing 11 214 locations and 32 236 samples). The remaining 20% of locations and samples were used to assess the predictive capacity of the models in held‐out data (hereafter the *testing set*: with 2804 locations and 6947 samples).

#### ErM fungal richness modeling

Data on species richness of ErM fungi were calculated as the number of ErM species present in a given sample. Then, we built a hierarchical model in the generalized linear modeling (GLM) framework and used Bayesian inference through the package brms (Bürkner, 2017) in R (R Core Team, 2023).

Since ErM fungal species richness is a count variable with excess zeros (43% of the samples had no ErM fungi), we modeled it using a zero‐inflated Poisson model (ZIP model hereafter). The ZIP model is a mixture of two processes: a Binomial process with logit link modeling the presence/absence of ErM fungi and a Poisson process with log link modeling the number of species in the samples given the presence of ErM fungi. As fixed explanatory variables, we included variables related to climate, soil chemistry and ErM vegetation. With the 19 bioclimatic variables retrieved from the CHELSA database, we explored pairwise correlations to exclude strongly correlated variables (Spearman correlation > 0.9). Eight bioclimatic variables were retained through this process: mean diurnal air temperature range (BIO2), isothermality (BIO3), mean daily air temperatures of the wettest quarter (BIO8), mean daily air temperatures of the driest quarter (BIO9), precipitation amount of the driest month (BIO14), precipitation seasonality (BIO15), mean monthly precipitation amount of the warmest quarter (BIO18), and mean monthly precipitation amount of the coldest quarter (BIO19). We also included pH, SOC and N as soil chemical descriptors, and ErM vegetation relative abundance as the sole vegetation descriptor. Variance inflation factors were < 5 for all variables used in modeling (Zuur *et al*., 2009). SOC, BIO14, BIO18, BIO19 and ErM vegetation relative abundance were log‐transformed. Quadratic transformations of bioclimatic and soil variables were also included in the models to allow nonlinear relationships. In addition to the environmental metadata, we also included log‐transformed sequencing depth to account for differences in sequencing effort across samples. All covariates were scaled to mean zero and unit variance. Then, we included a location‐level random effect to treat the samples with identical coordinates as repeated measures of the same locations, rather than independent observations of similar environmental conditions. Additionally, we included a continent‐level random effect as a proxy for broad‐scale residual spatial patterns in species richness. To quantify the importance of each group of explanatory variables (climate, soil, vegetation) in predicting ErM fungal species richness, we report each group's predictive capacity on the testing set using total and marginal *R* ^2^. Total *R* ^2^ represents the predictive capacity of a group when used as the sole set of predictors, whereas marginal *R* ^2^ represents its predictive capacity after accounting for the effects of the other groups. To calculate total predictive power, we computed the predictive power of models including variables from each of the three groups separately. To calculate marginal predictive power, we first computed the predictive power of the full model including all variables (climate + soil + vegetation). We then computed the predictive power of models including variables from two groups at a time (climate + soil, climate + vegetation, soil + vegetation). Each marginal predictive power was calculated by subtracting the predictive power of the two‐group model from that of the full model; for example, the marginal *R* ^2^ of climate = *R* ^2^(climate + soil + vegetation) − *R* ^2^(soil + vegetation). All models included the random effects and log‐transformed sequencing depth. To remove the influence of a given group when making predictions, we set its variables to their average values. We applied the same procedure to the relative abundance and species‐specific occurrence models described below.

We fitted the models assuming the default flat priors for the intercepts, but set narrower priors following a standard normal distribution for the slopes. We sampled the posterior distribution by running each of four Markov chains Monte Carlo (MCMC) for 3000 iterations including a burn‐in period of 1000 iterations. We ensured MCMC convergence by verifying that the potential scale reduction factors for the beta parameters obtained values close to one (Fig. S2).

#### ErM fungal relative abundance modeling

Data on the relative abundance of ErM fungi were calculated as the share of ITS sequences classified to ErM species divided by the total number of all fungal ITS sequences. Relative abundance data have the features of being bounded in [0, 1], being highly skewed and having excess zeros. To model such data, we implemented a zero‐inflated beta model (ZIB model hereafter) in the GLM framework through the R package brms (Bürkner, 2017). Previous studies have shown the suitability of this model to analyze compositional microbiome data (Chen & Li, 2016). The ZIB model is a mixture of two processes: a Binomial process with logit link modeling the presence/absence of ErM fungi in the samples, and a Beta process modeling the non‐zero relative abundances. We used the same set of fixed and random effects as in the ZIP model. The Beta distribution is parameterized using a mean parameter and a dispersion parameter. The mean parameter and the dispersion parameter were independently modeled with the same set of covariates; the mean parameter used a logit link and the dispersion parameter used a log link. Total and marginal predictive powers of different groups of explanatory variables were calculated as explained above, in the fungal richness modeling section.

We fitted the models and assessed MCMC convergence using the same procedures as for the richness models (Fig. S2).

#### ErM fungal species occurrence distribution modeling

To model the global occurrence patterns of ErM fungi at the species level, we implemented Hierarchical Modeling of Species Communities (Hmsc) using the R package Hmsc (Tikhonov *et al*., 2020) and the Hmsc‐hpc Python package (Rahman *et al*., 2024). Hmsc is a form of joint species distribution modeling (JSDM) (Warton, 2015) that models community data using a hierarchical Bayesian model in the GLM framework.

The response matrix Y (for notation, see Ovaskainen *et al*., 2017) consisted of the occurrence of 67 ErM fungal species (i.e. species occurring in at least 50 samples). To model the presence/absence of ErM fungi across the globe, we fitted a binomial model with a probit link function to each species. As fixed explanatory variables in the matrix X, we included the same variables as in the richness and relative abundance models, with their quadratic effects to allow intermediate optima along the gradients. Then, we included a location‐level random effect, implemented through latent factors (Ovaskainen *et al*., 2017), as well as a spatially explicit random effect to account for the influence of spatially structured processes such as dispersal limitation or omitted environmental factors. The broad range of spatial lags among locations (i.e. from a few meters to near 20 000 km) led to numerical instabilities when fitting the spatial model. Therefore, we clustered the locations into ‘islands’ using the dbscan() function from dbscan R package (Hahsler *et al*., 2019), setting the parameters so that the locations separated by *c*. 200 km were clustered into the same ‘island’ or cluster. Some clusters that encompassed excessively broad spatial ranges were further divided into clusters in a second step using the kmeans() function from the R package vegan (Oksanen *et al*., 2025). The whole procedure sorted the 14 977 locations into 316 clusters, with a minimum distance between clusters of 55 km (Table S2; Fig. S3). Then, we fitted a spatial random effect at the cluster level through spatially explicit latent variables (Ovaskainen *et al*., 2016). The spatially explicit random effect in Hmsc assumes that the site loadings of the latent variables (which model residual species occurrences and co‐occurrences) have exponentially decaying covariance structure. The number of latent factors used for each random effect in Hmsc is determined during model fitting and ranges between zero and the number of species in Y matrix (Ovaskainen & Abrego, 2020): the process ended with 13 latent factors for the location random effect and 10 for the spatial random effect. We excluded the prior probability of the scale parameters alpha for the spatial latent factors to alpha = 0, since the location random effect already captured location effects with no spatial dependence, and assumed the prior alpha ~ Uniform(148 km, 14 000 km) for the spatial random effect. Total and marginal predictive powers of different groups of explanatory variables were calculated as explained above, in the fungal richness modeling section. Additionally, to assess the relative contribution of each explanatory variable and random effect to the explained variance of each species in the Hmsc model, we used the custom function computeVariancePartitioning() from the R package Hmsc (Tikhonov *et al*., 2020).

We fitted the models assuming the default priors for the rest of the parameters and sampled the posterior distribution by running each of four MCMC chains for 3750 000 iterations including a burn‐in period of 1250 000 iterations. We thinned by 10 000 to obtain a total of 250 posterior samples per chain and 1000 posterior samples in total. We ensured MCMC convergence by verifying that the potential scale reduction factors for the beta parameters and alpha parameters obtained values close to one (Fig. S2).

#### Evaluation of model fit

The overall model fits of the ZIP model, ZIB model and Hmsc models were evaluated as the *R* ^2^ of the fit to the training set, as well as in terms of accuracy, discrimination power and calibration when predicting the testing set (Norberg *et al*., 2019). The fit was evaluated when predicting sample‐level species richness of the testing set and the species richness averaged over locations. To compare the Hmsc model with the ZIP, we summed all predicted probabilities of presence across the species to obtain an estimate of predicted species richness per sample (Calabrese *et al*., 2014). Accuracy was evaluated through the root mean square error (RMSE) between observed and predicted values; discrimination was evaluated through the Spearman correlation between the observed and predicted values; to evaluate calibration, we computed the interquartile range (IQR) of the predictions for each location and sample and calculated the proportion of the observed values that were contained within the IQR range.

Hmsc and ZIP models fitted well to the training data, although they slightly under‐predicted the highest observed richness values (Fig. S4). However, Hmsc performed better for accuracy, discrimination and calibration when predicting the testing set at both location and sample level (Table S3). The fit of the ZIB model to the training data was worse, particularly for the observations with a relative abundance > 0.2 (Fig. S4). However, those observations only represented *c*. 0.2% of the data. Better overall accuracy and discrimination power of the models when predicting location averages as compared to sample‐level values indicate that there was a significant variability between samples and within locations that the predictors of our models failed to capture, partly due to the lack of resolution of the databases at fine‐grain spatial scales (Table S3).

To assess the degree of the global environmental representativeness of our data, we performed a principal component analysis (PCA)‐based approach described in Averill *et al*. (2022) and van den Hoogen *et al*. (2019). First, we performed a PCA on observed data, using the set of covariates we used for modeling, and we used the centering values, the scaling values and the eigenvectors to transform the global covariate data into the same principal component space. Then, we created convex hulls for each of the bivariate combinations of the first six principal components, covering > 90% of the sample space variation. Using the coordinates of these convex hulls, we classified each global pixel as within or outside of the observed environmental conditions. Lastly, for the pixels that fell outside of the observed convex hull, we calculated the Euclidean distance between the convex hull and the pixel, thus calculating the degree of environmental extrapolation for each pixel in the globe.

#### Community composition analysis

To assess the main trends in community composition and associate them with global environmental gradients and spatial locations, we performed a principal coordinate analysis (PCoA) on the Bray–Curtis dissimilarity matrix of Hellinger‐transformed ErM fungal relative abundances. We performed the PCoA in three dimensions through the cmdscale() function from the vegan R package (Oksanen *et al*., 2025). Then, we used the envfit() function in the vegan R package to project all environmental variables used in modeling to the PCoA compositional space.

#### Correlations between soil organic carbon concentration and ErM fungal richness and relative abundance

To assess the global associations between soil organic carbon and ErM fungal richness, relative abundance and ErM vegetation relative abundance, we used soil organic carbon concentration aggregated across three depths: 0–5, 0–30 and 0–100 cm. These aggregated variables were derived from SoilGrids layers at depths 0–5, 5–15, 15–30, 30–60 and 60–100 cm, and computed as follows:

$$\mathrm{SO}C_{\mathrm{agg}}=\frac{\sum_{i=1}^{n}\mathrm{SO}C_{i}d_{i}}{\sum_{i=1}^{n}d_{i}}$$

For layers *i* with concentration SOC_*i*_ and thickness *d*_*i*_.

We considered three depth intervals to capture complementary aspects of soil carbon: 0–5 cm, matching the depth of fungal sampling; 0–30 cm, the standard topsoil layer used in global SOC assessments and most directly linked to surface processes (e.g. FAO GSOCmap; IPCC guidelines); and 0–100 cm, to approximate whole‐profile carbon while acknowledging that predictions at deeper horizons are more uncertain due to sparser data and stronger model dependence (Hengl *et al*., 2017).

#### Global fungal distribution maps

To build global fungal distribution maps, we used spatial grids with a resolution of 0.1666667 degrees by 0.1666667 degrees (latitude by longitude), which corresponds to *c*. 18.5 km by 18.5 km per grid cell at the equator. For the case of ErM fungal species richness, we extrapolated to the global grid using the Hmsc model, whereas to extrapolate the relative abundances, we used the ZIB model.

To build predicted species richness and relative abundance change maps under global change, we computed the model predictions of the above‐defined models using the bioclimatic variables under the ssp370 global change scenario for the period 2041–2070, and assuming the other covariates in their current values. Then, we subtracted the future richness and relative abundance values from the current values on a cell‐by‐cell basis, to build predicted change maps. Similarly, we predicted the future occurrence probabilities for each ErM fungal taxa; we transformed the occurrence probabilities to presences and absences by turning values larger than or equal to 0.5 to 1 and smaller than 0.5 to 0, and, lastly, computed the Sorensen dissimilarity between future and current communities on a cell‐by‐cell basis.

## Results

### Description of the dataset

Our analysis of ErM fungal species richness and distribution leveraged 39 163 soil samples from 551 independent studies, collated within the GlobalFungi Database v.5 (Větrovský *et al*., 2020). In total, 33 466 soil samples contained at least one ErM fungal species. Among the 107 species recognized as ErM fungi, 87 were present in at least one sample (Table S1), in total represented by > 13 million ITS rRNA sequences.

**Fig. 1:**
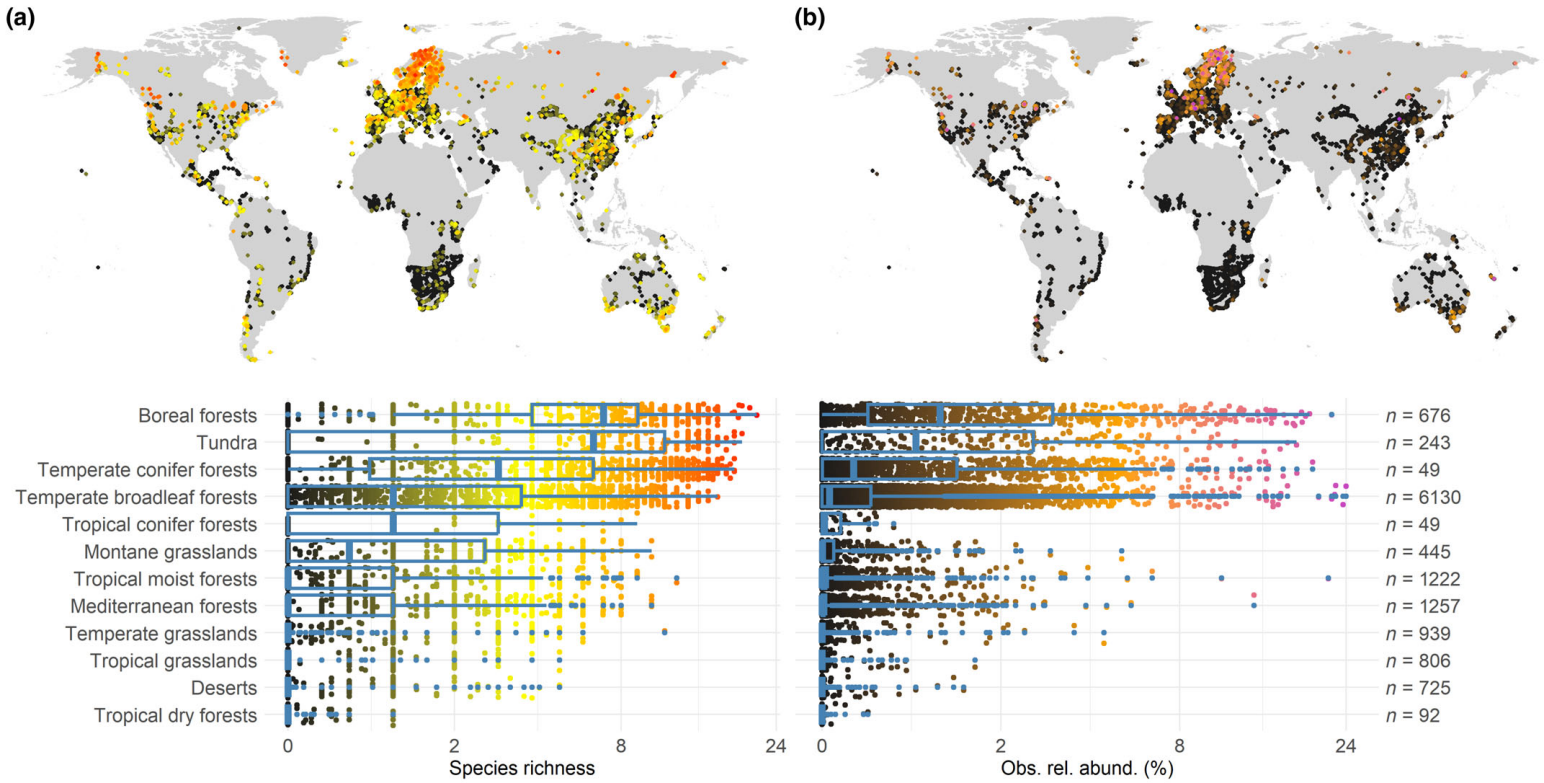
Sampled locations and observed richness and relative abundance of ericoid mycorrhizal fungi. (a) Global distribution of sampling locations and observed ericoid mycorrhizal fungal species richness by biomes. (b) Global distribution of sampling locations and observed relative abundance of ericoid mycorrhizal fungi by biomes. *X*‐axes of the bottom panels are in log scale for visualization. Boxplots indicate the median (center line), first and third quartiles (lower and upper box edges), and 1.5× IQR (box whiskers). The points in the maps and the boxplots are colored according to the species richness gradient (from black through yellow to red) and relative abundance gradient (from black through orange to purple). The numbers to the right of the bottom boxplots depict the number of locations representing each biome. Observed richness was corrected for differences in sequencing depth across samples by using a generalized linear model with a Poisson distribution, with species richness as the response variable and log‐transformed sequencing depth as the explanatory variable. Then, the model was used to predict species richness for each location by setting sequencing depth to its average value.

The most diverse ErM fungal communities were observed in high latitude biomes including tundra, boreal and temperate coniferous forests, with up to 20 species per sample (four species/sample on average; Fig. 1a). High species richness of ErM fungi was also observed in a few soil samples from tropical moist forest and Mediterranean forests, but overall richness of ErM fungi from these ecosystems was low (Fig. 1a). We found that ErM fungi were almost absent in tropical dry forests, grasslands and deserts. The relative proportion of ErM fungi within the soil mycobiome largely corresponded with their species richness (Figs 1b, S5). In samples from tundra and boreal and temperate forests, ErM fungi often represented > 10% of the fungal community. Using this dataset, we modeled the global ErM fungal species richness (ZIP and Hmsc models) and relative abundance (ZIB model), as well as the distribution of individual ErM fungal taxa (Hmsc model) in the soil mycobiome.

### Global patterns and environmental predictors of ericoid mycorrhizal fungal richness

**Fig. 2:**
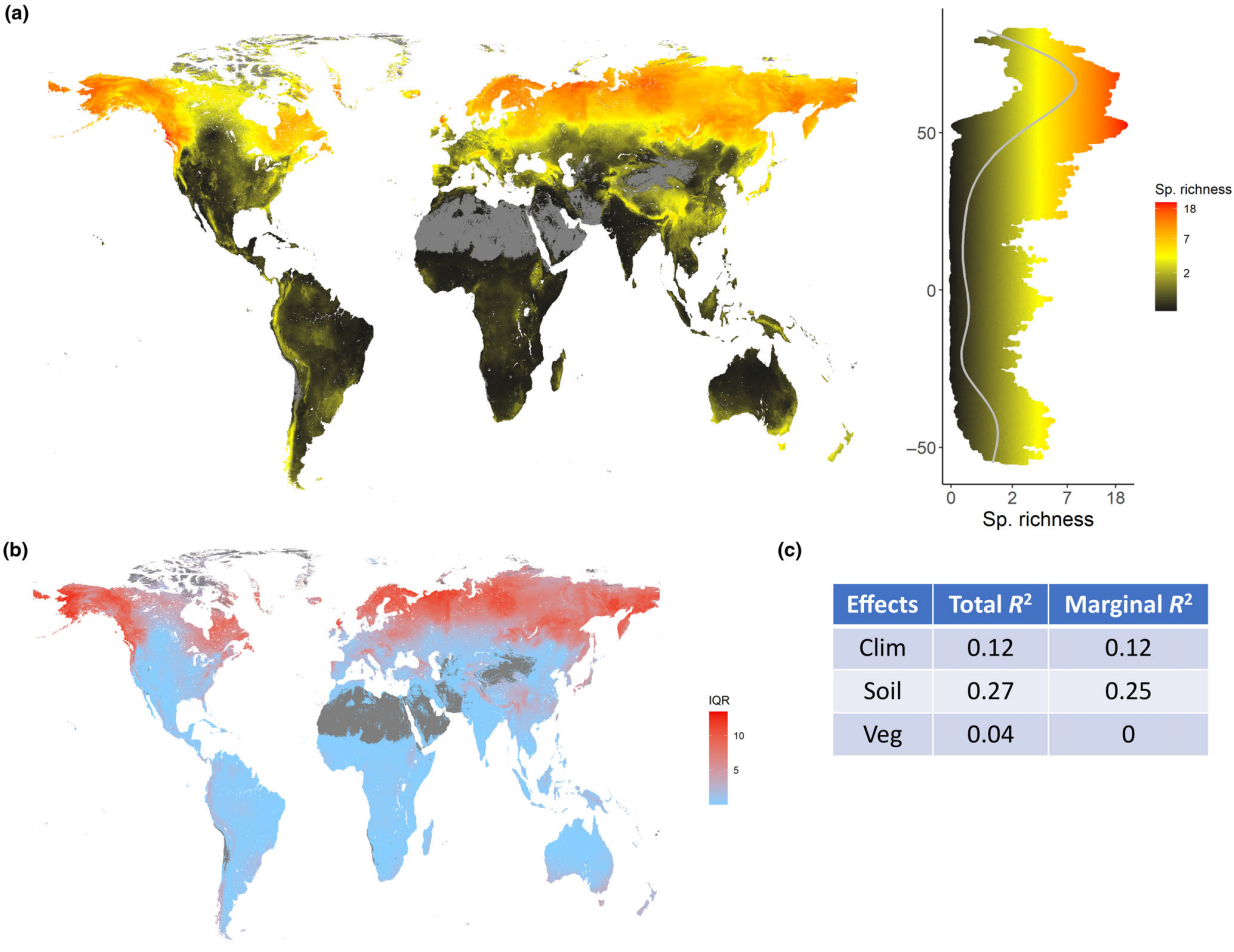
Global predictions of local ericoid mycorrhizal fungal richness, its latitudinal trends and environmental predictors. (a) Global predictions and latitudinal trends of ericoid mycorrhizal fungal species richness based on Hmsc model. (b) Model uncertainty of ericoid mycorrhizal fungal species richness predictions expressed as interquartile ranges of the posterior predicted values. Masked locations (gray) show sparsely vegetated zones and dense urban areas based on global land‐cover data. (c) Predictive capacity of richness of ericoid mycorrhizal fungal species of the *testing set*, using distinct groups of environmental variables. The total *R* ^2^ measures the predictive capacity of each group of variables (climate, soil chemistry and vegetation) when used as sole explanatory variables, whereas the marginal *R* ^2^ measures the predictive capacity of each group of variables after taking the effect of the other groups into account.

Our global models reveal a pronounced inverse latitudinal gradient in ErM fungal species richness (Fig. 2a). Predicted richness of ErM fungal species was particularly low in tropical lowlands and in the subtropics, but increased substantially toward the northern temperate regions, and this increase continued toward the boreal regions, where it peaked in the tundra. The global richness patterns were similar when predicting with the ZIP model, but exhibited sharper spatial variations due to the absence of a continuous spatial process in the model (Fig. S6). The highest recorded species richness came from very high latitudes (Fig. 1a), but the predictions of global diversity hotspots in northern Siberia and East Asia should be taken with some caution since these regions have been sampled only sparsely (Fig. S7) and the models identified them with sizable uncertainty (Fig. 2b).

Besides the clear latitudinal diversity pattern, our models also predict some potentially important azonal patterns in ErM fungal species richness. For instance, some mountainous regions of tropical moist forests are predicted to be local hotspots of ErM fungal species richness (Fig. 2a), which is likely driven by a subset of samples from these regions showing high ErM fungal species richness (Fig. 1a).

To identify the environmental factors contributing to the prediction of ErM fungal species richness, we divided the 12 explanatory variables into three groups: climate (eight variables), soil chemistry (three variables) and ErM vegetation relative abundance (one variable). We then quantified the predictive capacity of each group using total and marginal predictive capacity. Soil chemistry was the strongest predictor of ErM fungal species richness, followed by climate, based both on total and on marginal predictive power (Fig. 2c), with soil pH showing a strong negative marginal association with ErM fungal species richness (Figs S8, S9). Species richness tended to correlate with climate continentality, typical with high mean diurnal range (BIO2), low isothermality (BIO3) and with high precipitation in the growth season and high snow cover characterized by high precipitation of both the warmest (BIO18) and coldest (BIO19) quarters (Figs S8, S9).

Based on the Hmsc model, ErM vegetation relative abundance made only a minor contribution and showed no significant marginal predictive capacity once climate and soil chemistry were accounted for (Fig. 2c). By contrast, under the ZIP model, vegetation had a stronger total predictive power than climate and displayed a positive marginal predictive capacity – although weaker than climate – beyond the effects of climate and soil chemistry (Fig. S6). Moreover, ErM vegetation relative abundance exhibited a relatively strong positive marginal association with species richness according to the ZIP model (Figs S8, S9). The predictive capacity of vegetation for ErM species richness was weaker in the model with species‐specific responses (Hmsc) than in the community‐level model (ZIP).

### Global patterns and environmental predictors of ericoid mycorrhizal fungal relative abundance

**Fig. 3:**
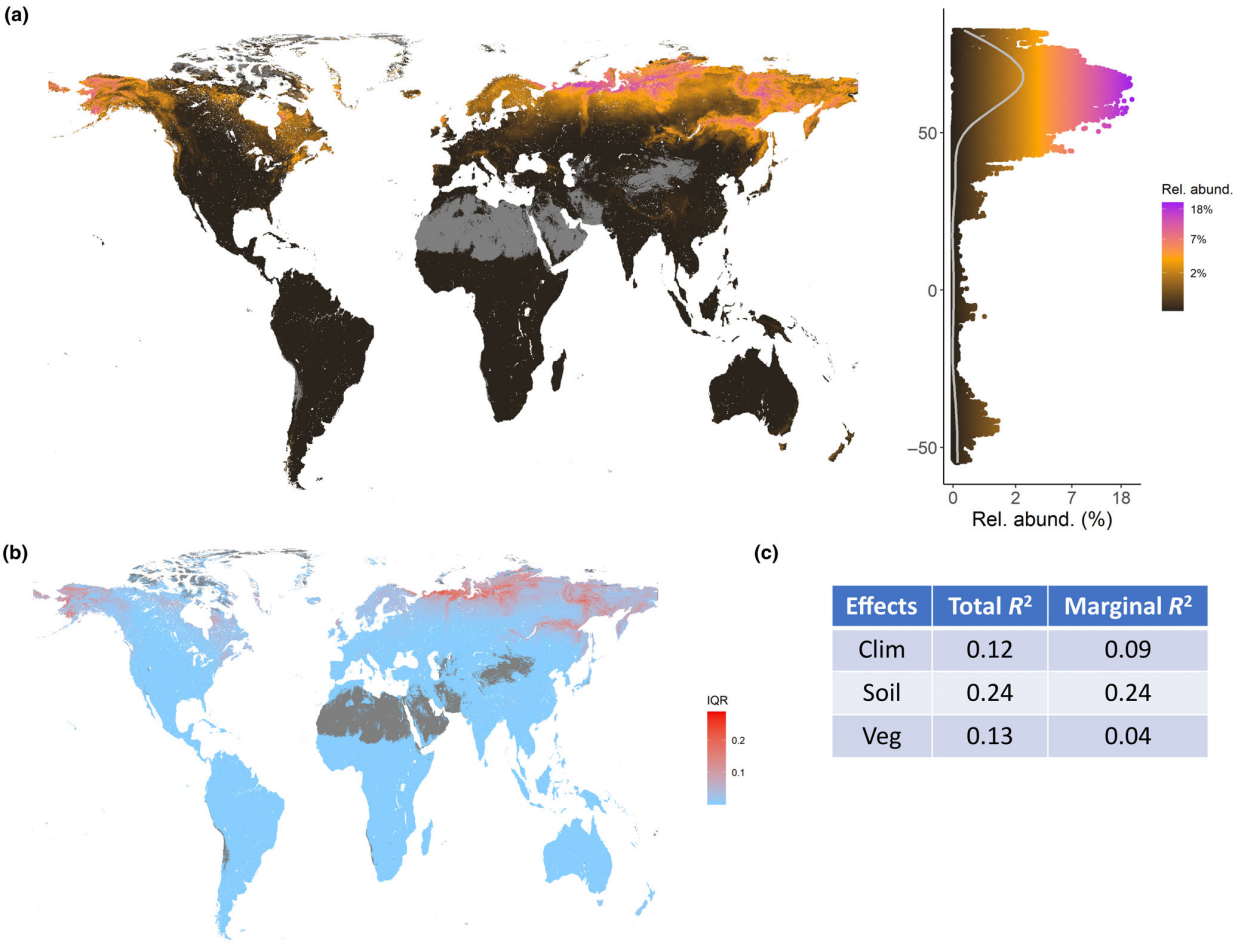
Global predictions of local relative abundance of ericoid mycorrhizal fungi, their latitudinal trends and environmental predictors. (a) Global predictions and latitudinal trends of ericoid mycorrhizal fungal relative abundance based on ZIB model. (b) Model uncertainty of ericoid mycorrhizal fungal relative abundance predictions expressed as interquartile ranges of the posterior predicted values. Masked locations (gray) show sparsely vegetated zones and dense urban areas based on global land‐cover data. (c) Predictive capacity of the relative abundance of ericoid mycorrhizal fungal species of the *testing set*, using distinct groups of environmental variables. The total *R* ^2^ measures the predictive capacity of each group of variables (climate, soil chemistry and vegetation) when used as sole explanatory variables, whereas the marginal *R* ^2^ measures the predictive capacity of each group of variables after taking the effect of the other groups into account.

Relative abundance of ErM fungi followed a global pattern largely similar to that of species richness, whereby the maximum relative abundance was centered at the highest northern latitudes (Fig. 3a), even though its estimates were associated with uncertainty (Fig. 3b). Although some samples originating from tropical regions harbored a relatively high number of ErM fungal species (Fig. 1a), the relative abundance of ErM fungi was very low (Fig. 1b).

Soil chemistry was the strongest predictor of ErM fungal relative abundance, with a pronounced negative marginal association with pH (Figs 3c, S10). Similar to the ZIP richness model, ErM vegetation relative abundance appeared as a stronger predictor of ErM fungal relative abundance than climate based on total predictive power; however, climate was a considerably stronger predictor based on the marginal predictive power (Fig. 3c), which indicates that an important part of the total effect of ErM vegetation was probably due to fungal and vegetation responses to climate and soil chemistry. Again, ErM vegetation relative abundance showed a clear positive marginal association with relative abundance of ErM fungi (Fig. S10).

### Ericoid mycorrhizal fungal community composition on a global scale

**Fig. 4:**
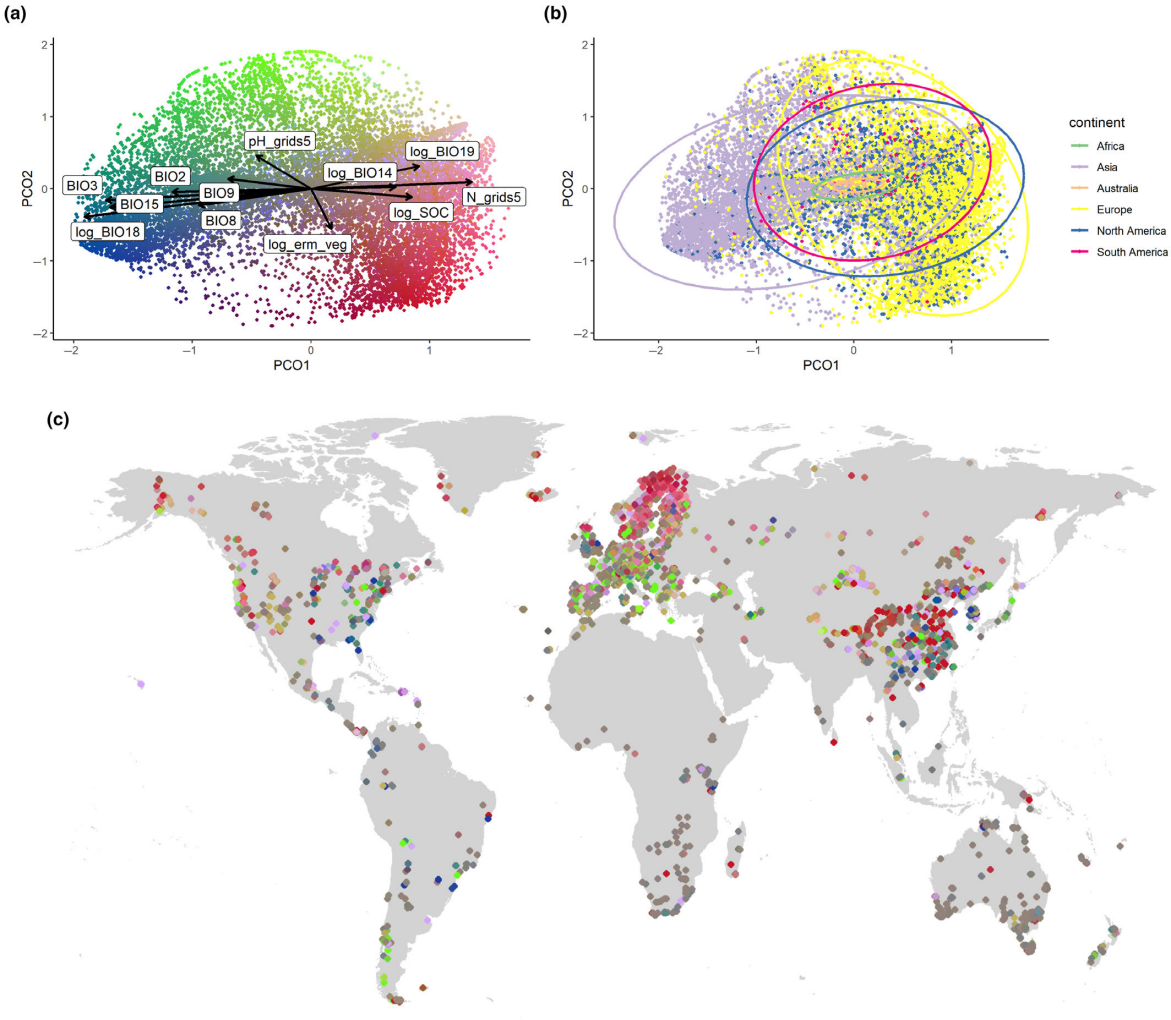
Global ericoid mycorrhizal fungal community composition. (a) Principal coordinate analysis (PCoA) ordination of ericoid mycorrhizal fungal community composition at the global scale, colored by the first three dimensions of the ordination transformed to an RGB color scale. All predictors used in modeling were passively projected onto the ordination space. (b) The same ordination as in (a), but colored by continents. (c) Global distribution of samples colored as in (a). BIO14, precipitation amount of the driest month; BIO15, precipitation seasonality; BIO18, mean monthly precipitation amount of the warmest quarter; BIO19, mean monthly precipitation amount of the coldest quarter; BIO2, mean diurnal air temperature range; BIO3, isothermality; BIO8, mean daily air temperatures of the wettest quarter; BIO9, mean daily air temperatures of the driest quarter.

To assess the main trends in the ErM fungal community composition and associate them with global environmental gradients and spatial locations, we performed a PCoA. The main axis of the ordination of ErM fungal community composition aligned with the environmental gradient from areas with low organic matter content, high precipitation in the warmest quarter, high isothermality and high precipitation seasonality to areas with opposite conditions with high organic matter content and high precipitation in the coldest quarter (Fig. 4a). By contrast, ErM fungal communities did not show a clear separation by continents (Fig. 4b), and generally, similar communities were observed in similar environmental conditions of different continents (Fig. 4c). In the same line, low levels of endemism were observed among ErM fungal species: more than half of the taxa were found on more than three continents, whereas only 20% were restricted to one or two continents; out of the taxa present in > 50 samples, only three were geographically limited to a single continent (Fig. S11).

**Fig. 5:**
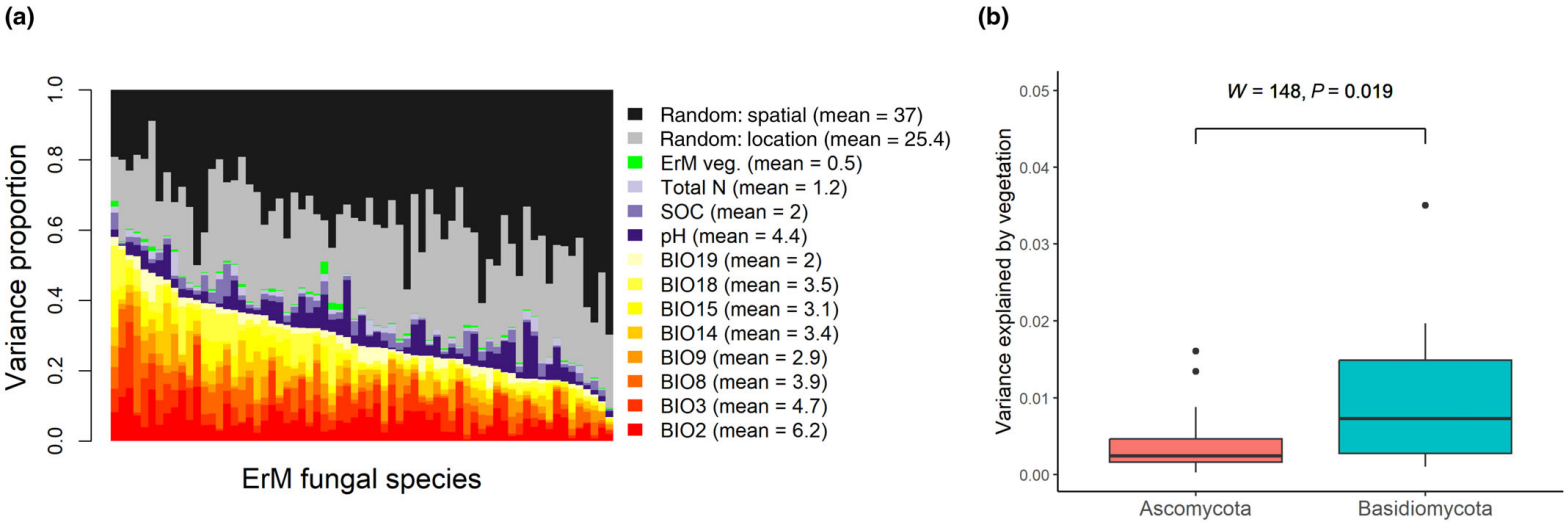
Variance partitioning of ericoid mycorrhizal (ErM) fungal species distribution modeling. (a) Variance partitioning of ErM species distribution modeling into environmental variables (fixed effects) and random effects. Values in the legend correspond to the proportion explained by each variable, averaged over all ErM fungal species. BIO14, precipitation amount of the driest month; BIO15, precipitation seasonality; BIO18, mean monthly precipitation amount of the warmest quarter; BIO19, mean monthly precipitation amount of the coldest quarter; BIO2, mean diurnal air temperature range; BIO3, isothermality; BIO8, mean daily air temperatures of the wettest quarter; BIO9, mean daily air temperatures of the driest quarter. (b) Comparison of the contribution of the relative abundance of ErM vegetation to the explained variance between basidiomycotan and ascomycotan ErM fungi. The null hypothesis of no difference between the two groups was tested using a Wilcoxon rank sum test.

From the explained variance in species occurrences, climatic variables captured the highest fraction (explaining on average 29.7% of the variance), followed by soil chemistry (explaining 7.6% of the variance), whereas ErM vegetation relative abundance only explained a minor fraction of the variance (0.5%) (Fig. 5a). Specifically, mean diurnal air temperature range was the primary predictor of distributions (Fig. 5), and most species showed a positive or hump‐shaped response (Fig. S12), indicating a tendency toward continental climate. The most important soil predictor was pH: Nearly all ErM fungal species exhibited a negative marginal association (Fig. S12). Although ErM vegetation explained little variance on average, it explained a significantly higher fraction of variance in basidiomycotan than in ascomycotan ErM fungi (Fig. 5b). Moreover, from 10 basidiomycotan species modeled in Hmsc, seven (70%) showed a significant (> 0.9 posterior statistical support) positive marginal association with ErM vegetation, whereas only 13 out of 56 (23%) modeled ErM fungal taxa from the Ascomycota showed a significant positive marginal association (Fig. S12).

Although ErM fungal communities showed little clustering on a global scale, the location and spatial random effects explained a very large proportion of the variance in ErM fungal species distributions in the Hmsc model (Fig. 5a), which indicates that ErM fungal distributions were strongly spatially structured from local to intermediate spatial scales (the scale of the leading spatial latent factor was *c*. 1600 km).

### Ericoid mycorrhizal fungal connections to soil carbon and predicted climate change responses

**Fig. 6:**
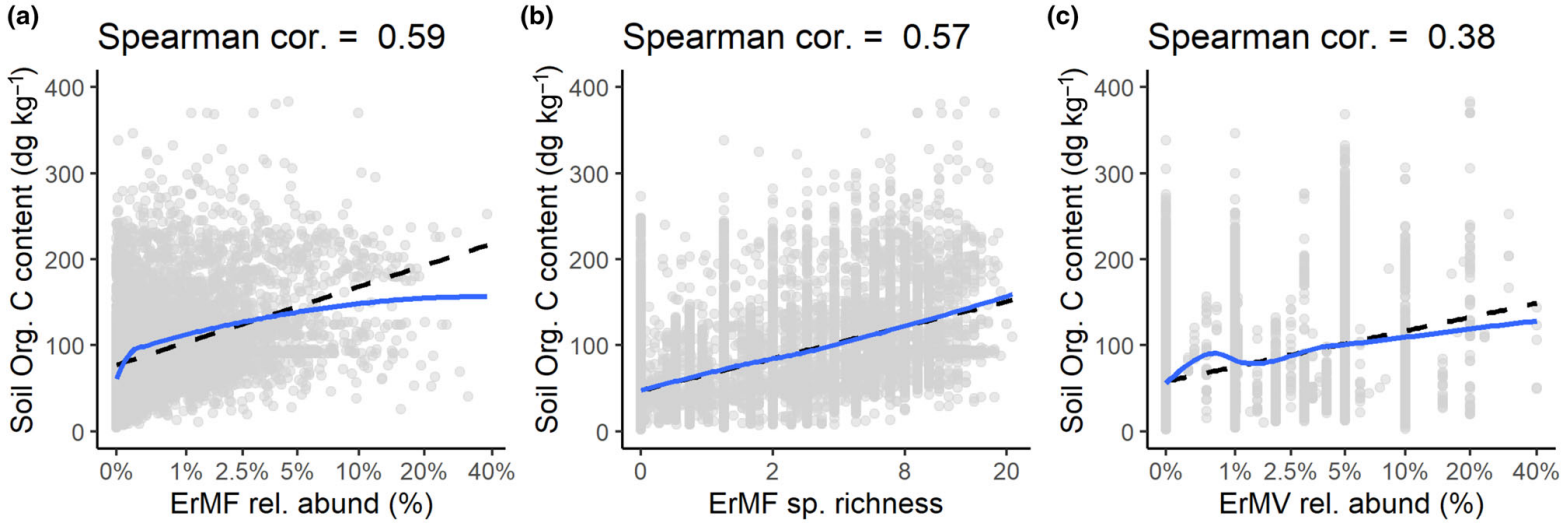
Correlation of soil organic carbon content with ericoid mycorrhizal fungi (ErMF) and ericoid mycorrhizal vegetation (ErMV). Correlations between global soil organic carbon content and (a) ericoid mycorrhizal fungal relative abundance, (b) ericoid mycorrhizal fungal species richness and (c) ericoid mycorrhizal vegetation relative abundance. The dashed black line captures the linear relationship, whereas the blue smooth line corresponds to a Locally Estimated Scatterplot Smoothing (LOESS) fit. Note the log scale of the *x*‐axes.

The observed relative abundance of ErM fungi, similar to observed species richness, was both positively correlated with surface SOC at the global scale (Fig. 6a,b), slightly stronger than the percentage of ErM plants in vegetation cover (Soudzilovskaia *et al*., 2019; Fig. 6c). As a sensitivity analysis, we also ran similar correlations with SOC at 0–30 cm and 0–100 cm depths obtaining very similar results (Fig. S13).

**Fig. 7:**
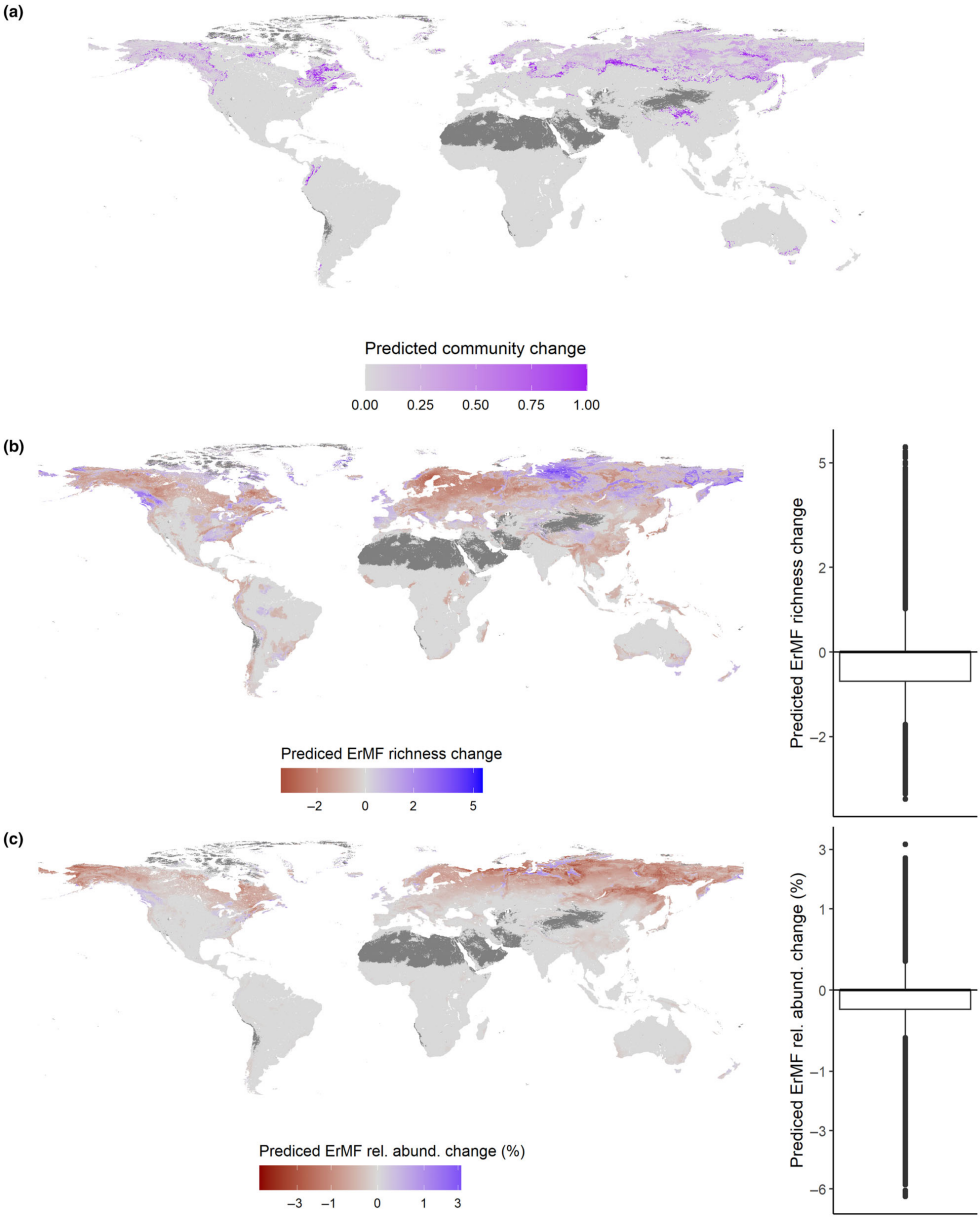
Predicted change in the local species richness and relative abundance of ericoid mycorrhizal fungi (ErMF) under the global change SSP370 scenario for the 2041–2070 period. (a) Predicted community composition change in ErMF under global change, expressed as Sorensen dissimilarity between current and predicted future communities. (b) Predicted change in ErMF species richness under global change. The value indicates the number of species gained/lost. The contiguous boxplot also shows the expected change in species numbers at each global location, indicating that the map is dominated by species losses. (c) Predicted absolute gain/loss of ErMF relative abundance under global change. The contiguous boxplot also shows the predicted change in relative abundance at each global location, indicating that the map is dominated by relative abundance losses. Predicted richness and relative abundance changes are in square‐root scale for better visualization. Masked locations (gray) show sparsely vegetated zones and dense urban areas based on global land‐cover data.

Considering the potential importance of ErM fungi for soil C sequestration in areas with large and sensitive soil organic carbon pools, we used the above models to make global predictions for climatic conditions under the SSP370 global change scenario for the period 2041–2070. Subsequently, we calculated the expected magnitudes of ErM fungal community turnover, as well as changes in richness and relative abundance, between current and future climatic conditions. For the purposes of this comparison, we assume that soil chemistry and ErM vegetation relative abundance remain constant and ErM fungi have little to no dispersal limitation (which may be fairly accurate given that some individual ErM fungal species seem to have broad distributions; Fig. S14). Under these considerations, expected species turnover is predicted to be highest in areas with high diversity and relative abundance of ErM fungi (Fig. 7a). The turnover is primarily driven by predicted losses in ErM fungal species richness, with 32% of global locations expected to lose species and 26% predicted to gain species (Fig. 7b). Similarly, ErM fungal relative abundance is projected to decline in 38% of global locations, while only 4% are predicted to experience gains (Fig. 7c).

## Discussion

This study provides empirical evidence on the global distribution of ErM fungal richness, helping to fill a key gap needed to evaluate a proposed three‐belt pattern of mycorrhizal fungal diversity (Read, 1991): ErM fungi peaking in tundra regions, ectomycorrhizal (EcM) fungi in temperate and boreal regions, and AM fungi in the tropics. We find a strong inverse latitudinal gradient in ErM fungal richness, with richness peaking at northern latitudes between 60° and 70°. Such a biogeographic pattern contrasts with the latitudinal diversity gradients reported for most macroorganisms (e.g. plants, invertebrates, birds, and mammals; Brown, 2014), but has been occasionally observed in microorganisms (Ladau *et al*., 2013; Tedersoo *et al*., 2014).

Van Nuland *et al*. (2025) recently reported that AM fungal richness is highest in tropical and subtropical regions, whereas EcM fungal richness peaks at northern latitudes *c*. 50°. Together with our results, these findings support the three‐belt pattern in which richness peaks of the three major mycorrhizal types (AM, EcM and ErM fungi) are systematically offset along latitudinal gradients. This pattern aligns with shifts in the dominance of corresponding mycorrhizal vegetation types, with AM vegetation prevailing in tropical regions, EcM vegetation in temperate zones, and ErM vegetation in high‐latitude environments (Soudzilovskaia *et al*., 2019; Steidinger *et al*., 2019). In line with the recent results of Van Nuland *et al*. (2025), our study corroborates that regions harboring the highest mycorrhizal fungal species richness do not mutually correspond among the three dominant mycorrhizal types. The limited correspondence poses a potential challenge for future conservation efforts to comprehensively safeguard the biodiversity of plant‐symbiotic fungi.

In addition to the inverse latitudinal pattern, our models predict local hotspots of ErM fungal species richness in mountainous regions of tropical moist forests. Tropical mountain systems such as the Andes, New Guinea Highlands and the Himalayas host a high diversity of ErM plants (Kohout, 2017), which may promote the occurrence of ErM fungi. Previous studies in these regions have primarily characterized fungal communities associated with the roots of ErM plants (e.g. Kohout, 2017; Mujica *et al*., 2024; Luo *et al*., 2025) but have been limited in their ability to identify novel ErM taxa due to methodological constraints, particularly the lack of resynthesis experiments required to confirm the ErM lifestyle (Vohník, 2020). Although we detected numerous recognized ErM fungi in tropical regions, it is likely that tropical Ericaceae host a broader diversity of ErM fungi, including undescribed taxa. Consequently, our estimates may underestimate local ErM fungal species richness. By contrast, the predicted relative abundance of ErM fungi was low in tropical mountain regions identified as species‐rich. This discrepancy may reflect the structure of ErM vegetation in these systems: Although some tropical mountains are hotspots of ErM plant diversity (Kohout, 2017), these plants are often epiphytic and lack direct contact with soil (Setaro & Kron, 2011; Mujica *et al*., 2024), and they rarely dominate local vegetation (Kron *et al*., 2002). By contrast, ericoid dwarf shrubs frequently dominate tundra communities and the understory of boreal and temperate coniferous forests (Kohout, 2017; Ward *et al*., 2022).

Although ErM fungal diversity hotspots occur in northern tundra, where ericoid dwarf shrubs dominate the local vegetation (Bueno *et al*., 2017; Soudzilovskaia *et al*., 2019), our results on the relationship of ErM fungi with their host plants are complex. The strength of association ranged from very weak to moderate, depending on whether we analyzed species occurrence, richness or relative abundance, and on whether environmental confounders were included. The weakest effects were observed for individual taxon occurrences, suggesting that many ErM fungal taxa have limited dependence on their host plants. By contrast, greater ErM vegetation relative abundance was associated with higher overall ErM fungal richness and relative abundance. This pattern is consistent with the relatively flexible life strategies of ErM fungi, which can grow as free‐living saprotrophs (Martino *et al*., 2018) or as endophytes in roots of various plant species (Chambers *et al*., 2008), and thus show low dependence on a strictly mycorrhizal lifestyle (Põlme *et al*., 2018; Martin & van der Heijden, 2024). However, the stronger association observed for basidiomycotan ErM fungi from the Sebacinales order indicates that this group might exhibit a more obligatory biotrophic lifestyle compared to ascomycotan ErM fungi, from the Helotiales, as recently suggested by Mielke *et al*. (2025).

The weak marginal predictive power of ErM vegetation relative abundance after accounting for environmental predictors suggests that ErM fungal richness may be driven more by soil conditions characteristic of ErM vegetation (e.g. low pH) than by host plant abundance *per se*. However, this interpretation is not mutually exclusive with indirect plant‐mediated effects: Part of the environmental signal may still operate through host plants. For example, the height of ericoid shrubs in boreal forests and tundra is strongly influenced by winter snow cover, which in turn affects their biomass and productivity during the growing season (Bienau *et al*., 2014). Moreover, because we used ErM vegetation relative abundance as the sole descriptor of ErM vegetation, we did not capture potential diversity or compositional effects and likely underestimated the overall contribution of ErM vegetation to fungal patterns.

The low level of endemism and weak continental pattern observed in our study suggest a high dispersal capacity of ErM fungi at the global scale. In contrast to macroorganisms such as vertebrates and plants, the distribution of fungi is generally considered to be less constrained by geographic barriers, probably due to their better dispersal capacity (Roper *et al*., 2010; Větrovský *et al*., 2019). However, the geographic constraints and the levels of endemism differ among fungal taxonomic and ecological groups. While obligatory symbiotic EcM fungi tend to be more geographically limited (Peay *et al*., 2007; Tedersoo *et al*., 2022; Abrego *et al*., 2024), facultatively symbiotic ErM fungi might experience lower dispersal limitations (Ward *et al*., 2022), since they can thrive in soils even in the absence of their potential host plants (Bergero *et al*., 2003; Vohník *et al*., 2013). Therefore, the global distributions of ErM fungal species seem to be governed primarily by environmental conditions, with little to no evidence for strong limitation by dispersal. Nevertheless, our reliance on molecularly defined Species Hypotheses as a proxy for fungal species warrants caution when interpreting patterns of endemicity. Although Species Hypotheses are widely used for molecular identification, they do not necessarily correspond to taxonomically described species, let alone to biological species (Noffsinger *et al*., 2025).

On the other hand, we did observe marked spatial structure in ErM fungal species distributions at local and regional scales. Many ecological processes might have contributed to the spatial structure in the residual ErM fungal species occurrences and co‐occurrences at these scales, including local and regional colonization‐extinction dynamics (Leibold *et al*., 2004), biotic interactions among species (Kneitel & Chase, 2004), anthropogenic influences or spatially structured environmental variables that were not available to inform our models. Since the climatic, soil and vegetation variables used for modeling were retrieved from global databases that may fail to capture local variation of environmental conditions, the random effects of the models might also have captured residual patterns derived from such biases. Regardless, our data indicate that ErM fungal species distributions are less geographically restricted at the global scale than those of other fungal guilds, particularly compared with EcM fungi.

We found moderate to strong correlations between soil organic carbon concentration and ErM fungal richness and relative abundance across multiple soil depths. The potential role of ErM symbioses in soil carbon sequestration has been noted previously (Clemmensen *et al*., 2013, 2015), as both ErM plants and fungi can contribute to SOC accumulation through the production of secondary compounds in plant and fungal tissues (Ward *et al*., 2022). In particular, the presence of melanin in ErM fungal cell walls may increase the recalcitrance of fungal necromass (Fernandez *et al*., 2019; Maillard *et al*., 2023). However, these results are based on correlation analyses and are likely influenced by multiple confounding factors. Further studies are therefore required to assess whether ErM fungi play a causal role in soil carbon storage.

Our results forecast a decline in ErM fungal richness and relative abundance under the SSP370 global change scenario for the period 2041–2070. Similar to recent fungal climate change studies (Qin *et al*., 2023; Van Nuland *et al*., 2024), our models indicate northward shifts in climatically suitable conditions, moving from areas currently occupied by boreal forests toward the present tundra. Sites with the largest predicted declines in ErM fungal diversity and relative abundance correspond to the regions where climate‐driven decline in soil carbon is expected to be highest (Crowther *et al*., 2016). Guerra *et al*. (2022) identified high latitudes of the Northern Hemisphere as hot spots of soil microbial‐driven ecosystem services, such as carbon sequestration, decomposition or pest control. Many of these areas (e.g. high latitudes of Europe and Asia, northeast of North America) largely overlap with areas predicted to experience a significant decline in ErM fungal species richness and relative abundance, although these predictions are associated with higher model uncertainty. This highlights the importance of gathering more information on ErM symbioses from these critically under‐sampled environments to improve model accuracy and to more fully understand the role of ErM fungi in building and maintaining some of the Earth's largest soil C stocks.

### Conclusion

This study provides the missing empirical evidence for ErM fungal distribution that fills a gap needed to support a three‐belt pattern of mycorrhizal fungal species richness on a global scale: ErM fungi reaching their highest richness in the tundra, ectomycorrhizal fungi in temperate and boreal regions, and AM fungi in the tropics. The limited correspondence of the regions harboring the highest mycorrhizal fungal species richness poses a potential challenge for future conservation efforts. Both the species richness and relative abundance of ErM fungi are strongly correlated with soil organic carbon content on a global scale. Furthermore, we demonstrate that climate change will reduce ErM fungal abundance across 38% of the land cover of their current global distribution, with possible consequences for vital ecosystem processes related to soil carbon cycling.

## Competing interests

None declared.

## Author contributions

IO and PK designed the study. TV, PB, CT and PK made the data curation. IO, FB, PTD, MEVN, CQ and PK analyzed the data. All authors contributed to the data interpretation. IO and PK wrote the original draft. FB, AM, MEVN, CQ, TK, NAS and PB reviewed the first draft. All the authors reviewed and contributed to the final version of the manuscript.

## Disclaimer

The New Phytologist Foundation remains neutral with regard to jurisdictional claims in maps and in any institutional affiliations.

## Supporting information


**Fig. S1** Species accumulation curves by continents as a function of the number of sampling locations considered.
**Fig. S2** Mixing statistics evaluating the ZIP, ZIB and Hmsc model fit.
**Fig. S3** Global map indicating the sampling locations in black and spatial clusters in red.
**Fig. S4** Model fit in the training set of the ZIP, ZIB and Hmsc models.
**Fig. S5** Correlation between ericoid mycorrhizal fungal relative abundance and ericoid mycorrhizal fungal species richness.
**Fig. S6** Global predictions and latitudinal trends of ericoid mycorrhizal fungal species richness based on the ZIP model.
**Fig. S7** Map of environmental representativeness of the Global Fungi 5 database.
**Fig. S8** Marginal associations of explanatory variables with ericoid mycorrhizal fungal species richness, computed from the Hmsc model.
**Fig. S9** Marginal associations of explanatory variables with ericoid mycorrhizal fungal species richness, computed from the ZIP model.
**Fig. S10** Marginal associations of explanatory variables with ericoid mycorrhizal fungal relative abundance, computed from the ZIB model.
**Fig. S11** Distribution of the number of occurrences of species hypotheses in different numbers of continents.
**Fig. S12** Marginal associations of explanatory variables with ericoid mycorrhizal fungal species occurrences, computed from the Hmsc model.
**Fig. S13** Correlations between global soil organic carbon content, and ericoid mycorrhizal fungal relative abundance, ericoid mycorrhizal fungal species richness and ericoid mycorrhizal vegetation relative abundance.
**Fig. S14** Global distribution maps of common ericoid mycorrhizal fungal taxa.

**Table S1** List of ericoid mycorrhizal fungal taxa.
**Table S2** List of sampling sites with metadata.
**Table S3** Evaluation of model fit in testing set in terms of root mean square error, Spearman correlation and % of observed values within the prediction interquartile range.
Please note: Wiley is not responsible for the content or functionality of any Supporting Information supplied by the authors. Any queries (other than missing material) should be directed to the *New Phytologist* Central Office.

## Data Availability

All data used in this study are available in the original papers, and the compiled dataset is available on https://globalfungi.com. Sufficient data and R scripts to reproduce the modeling work of this study are accessible via the Zenodo repository at doi: 10.5281/zenodo.17746133.
