# Supplementary material for: Global distribution and biogeography of ericoid mycorrhizal fungi
Source: New Phytol. 2026 Jun 12;251(4):2135–51. doi: 10.1111/nph.71334 (PMC13373864; doi:10.1111/nph.71334)
Supplement: Supplementary file 1 — Fig. S1 Species accumulation curves by continents as a function of the number of sampling locations considered. Fig. S2 Mixing statistics evaluating the ZIP, ZIB and Hmsc model fit. Fig. S3 Global map indicating the sampling locations in black and spatial clusters in red. Fig. S4 Model fit in the training set of the ZIP, ZIB and Hmsc models. Fig. S5 Correlation between ericoid mycorrhizal fungal relative abundance and ericoid mycorrhizal fungal species richness. Fig. S6 Global predictions and latitudinal trends of ericoid mycorrhizal fungal species richness based on the ZIP model. Fig. S7 Map of environmental representativeness of the Global Fungi 5 database. Fig. S8 Marginal associations of explanatory variables with ericoid mycorrhizal fungal species richness, computed from the Hmsc model. Fig. S9 Marginal associations of explanatory variables with ericoid mycorrhizal fungal species richness, computed from the ZIP model. Fig. S10 Marginal associations of explanatory variables with ericoid mycorrhizal fungal relative abundance, computed from the ZIB model. Fig. S11 Distribution of the number of occurrences of species hypotheses in different numbers of continents. Fig. S12 Marginal associations of explanatory variables with ericoid mycorrhizal fungal species occurrences, computed from the Hmsc model. Fig. S13 Correlations between global soil organic carbon content, and ericoid mycorrhizal fungal relative abundance, ericoid mycorrhizal fungal species richness and ericoid mycorrhizal vegetation relative abundance. Fig. S14 Global distribution maps of common ericoid mycorrhizal fungal taxa. [file NPH-251-2135-s002.docx]

New Phytologist Supporting Information

Article title: Global distribution and biogeography of ericoid mycorrhizal fungi

Authors: Iñaki Odriozola^1,2^, Tomáš Větrovský^1^, Florian Barbi^1^, Antonín Machac^1^, Priscila Thiago Dobbler^1^, Cristina Turcu^1,3^, Michael E. Van Nuland^2^, Clara Qin^2^, Toby Kiers^2,4^, Nadejda A. Soudzilovskaia^5^, Petr Baldrian^1^, Petr Kohout^1,3^

1) Institute of Microbiology of the Czech Academy of Sciences, Vídeňská 1083, 142 20 Prague, Czechia

2) Society for the Protection of Underground Networks (SPUN), 3500 South DuPont Highway Suite EI-101, Dover, DE 19901, USA

3) Faculty of Science, Charles University, Albertov 6, 128 00 Prague, Czechia

4) Amsterdam Institute for Life and Environment (A-LIFE), Section Ecology & Evolution, Vrije Universiteit Amsterdam, Van der Boechorststraat 3, 1081 BT Amsterdam, the Netherlands

5) Centre for Environmental Sciences, Hasselt University, Martelarenlaan 42, 3500 Hasselt, Belgium

Article acceptance date: 5 May 2026


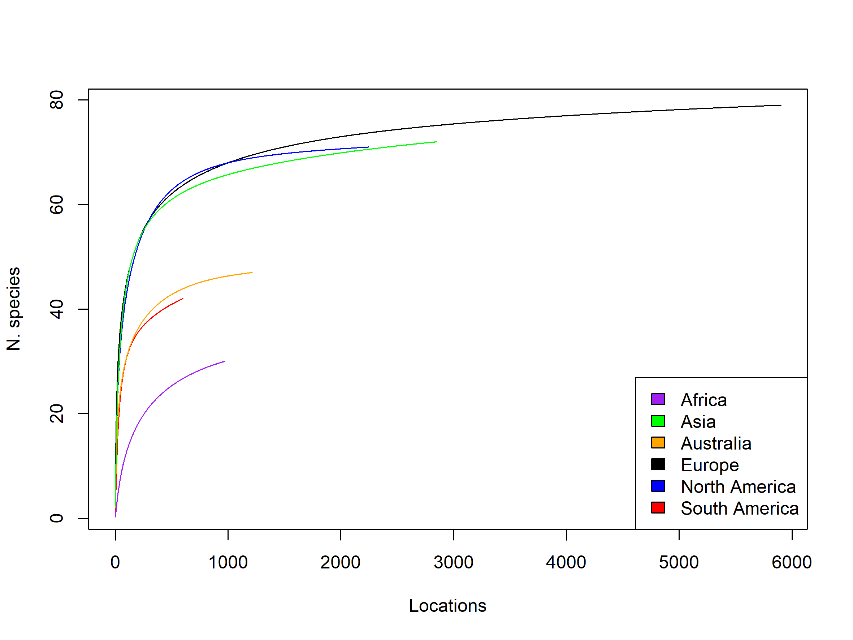


**Fig. S1.** Species accumulation curves by continents as a function of number of sampling locations considered.


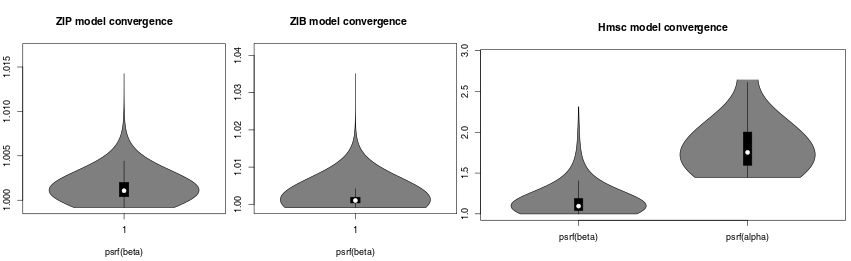


**Fig. S2.** Mixing statistics evaluating ZIP, ZIB and Hmsc model fit. Values depict potential scale reduction factors (psrf) for beta parameters in the case of ZIP and ZIB models and, beta and alpha parameters in the case of the Hmsc model.


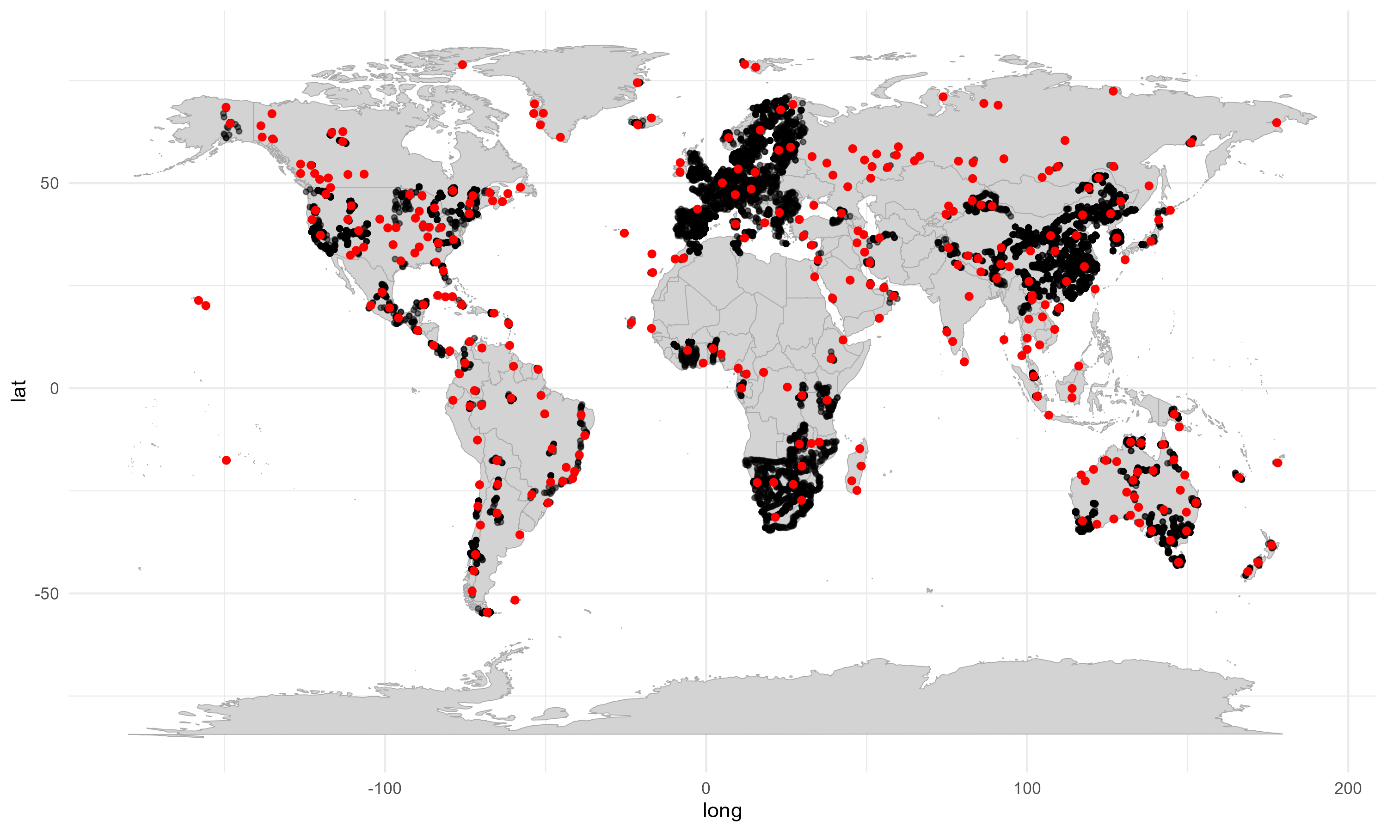


**Fig. S3.** Global map indicating the sampling locations in black and spatial clusters in red.


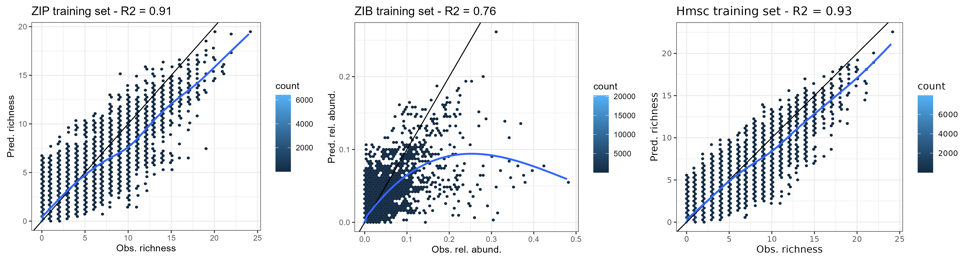


**Fig. S4.** Model fit in the training set of the ZIP, ZIB and Hmsc models.


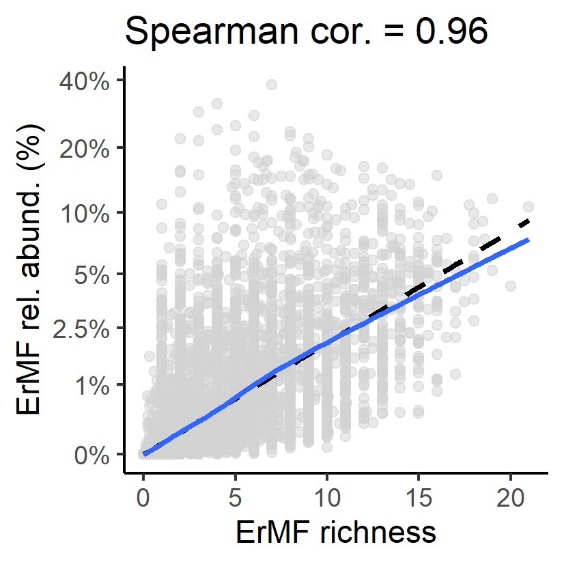


**Fig. S5.** Correlation between ericoid mycorrhizal fungal relative abundance and ericoid mycorrhizal fungal species richness. Dashed black line captures the linear relationship whereas the blue smooth line corresponds to a LOESS fit. Note the log scale of the y-axis.


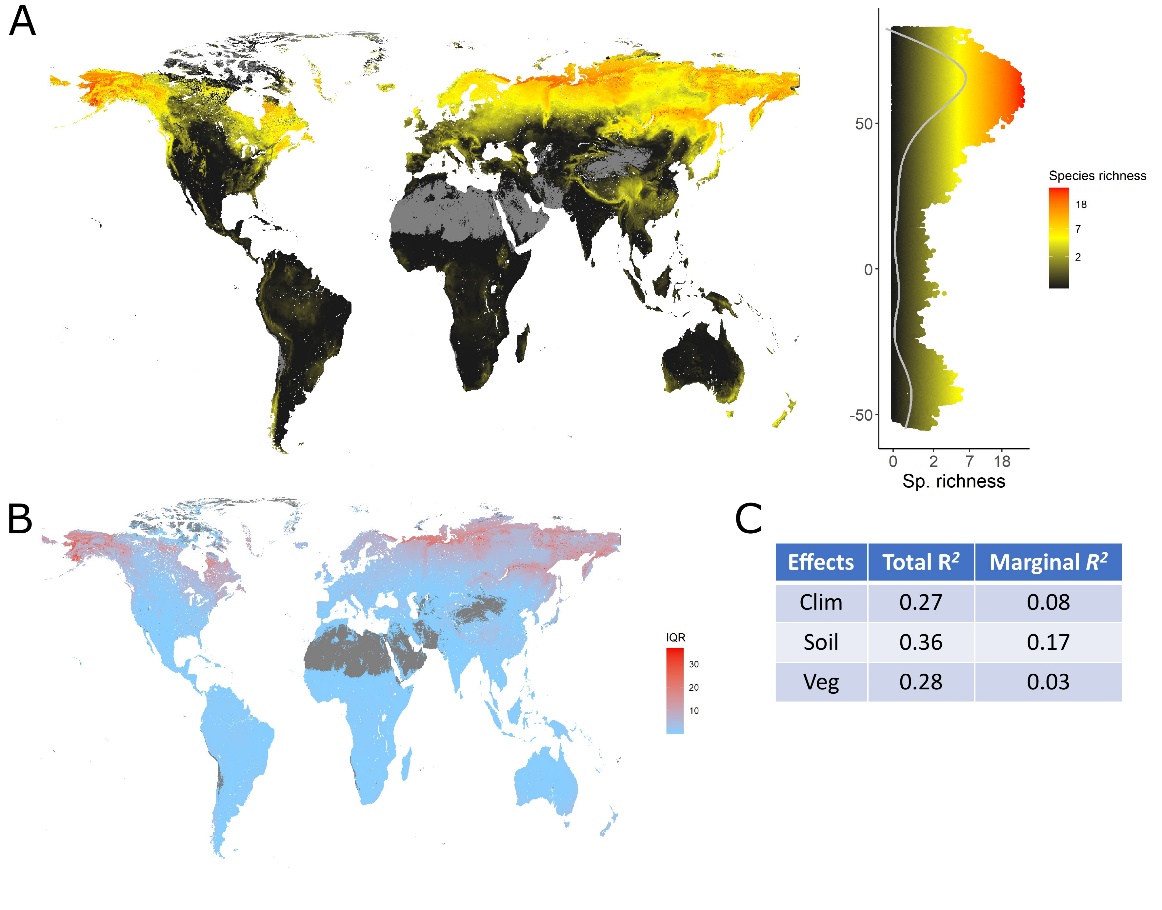


**Fig. S6.** A) Global predictions and latitudinal trends of ericoid mycorrhizal fungal species richness based on ZIP model. B) Model uncertainty of ericoid mycorrhizal fungal species richness predictions expressed as inter-quartile ranges of the posterior predicted values. Masked locations (grey) show sparsely vegetated zones and dense urban areas based on global land-cover data. C) Predictive capacity of richness of ericoid mycorrhizal fungal species of the *testing set*, using distinct groups of environmental variables. The total *R^2^* measures the predictive capacity of each group of variables (climate, soil chemistry and vegetation) when used as sole explanatory variables, whereas the marginal *R^2^* measures the predictive capacity of each group of variables after taking the effect of the other groups into account.


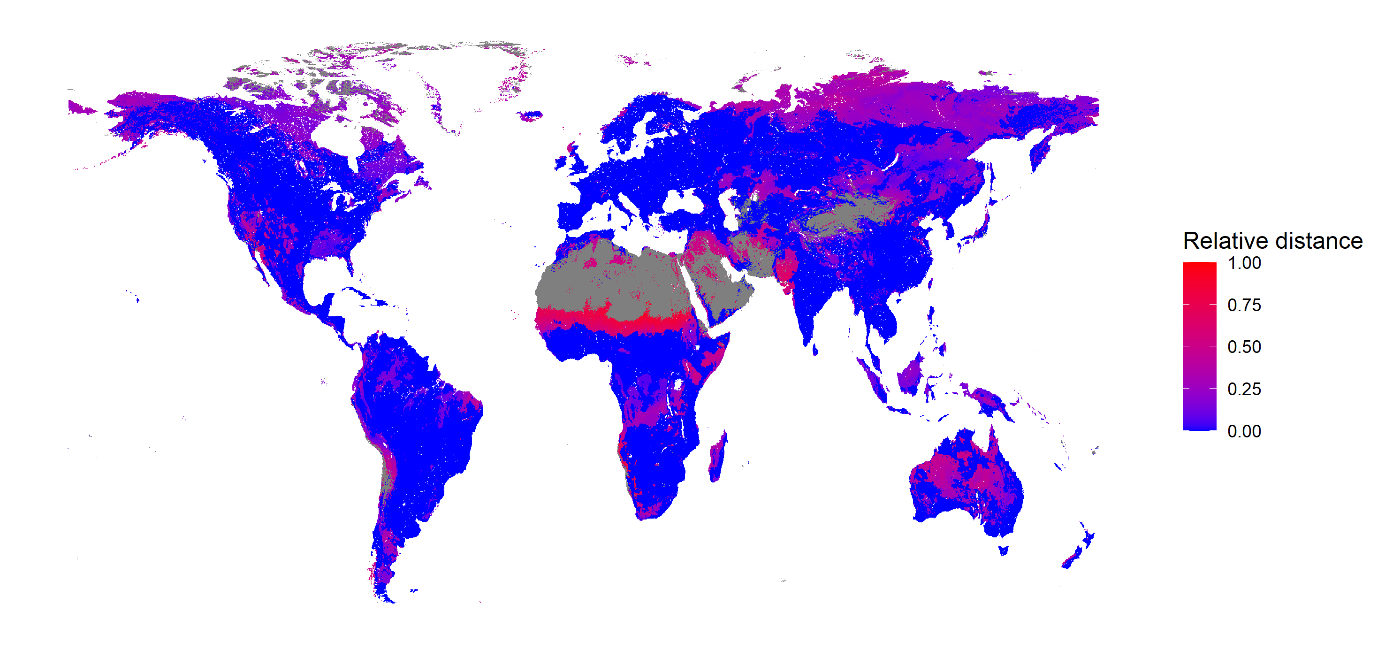


**Fig. S7.** Map of environmental representativeness of Global Fungi 5 database. Color represents the distance of local environmental conditions from the closest samples present in the database. Blue color indicates environmental conditions represented by sample locations; the gradient towards red indicates increasing relative distance between the environmental conditions in the pixel and the environmental conditions of the observed data. Masked locations (grey) show sparsely vegetated zones and dense urban areas based on global land-cover data.


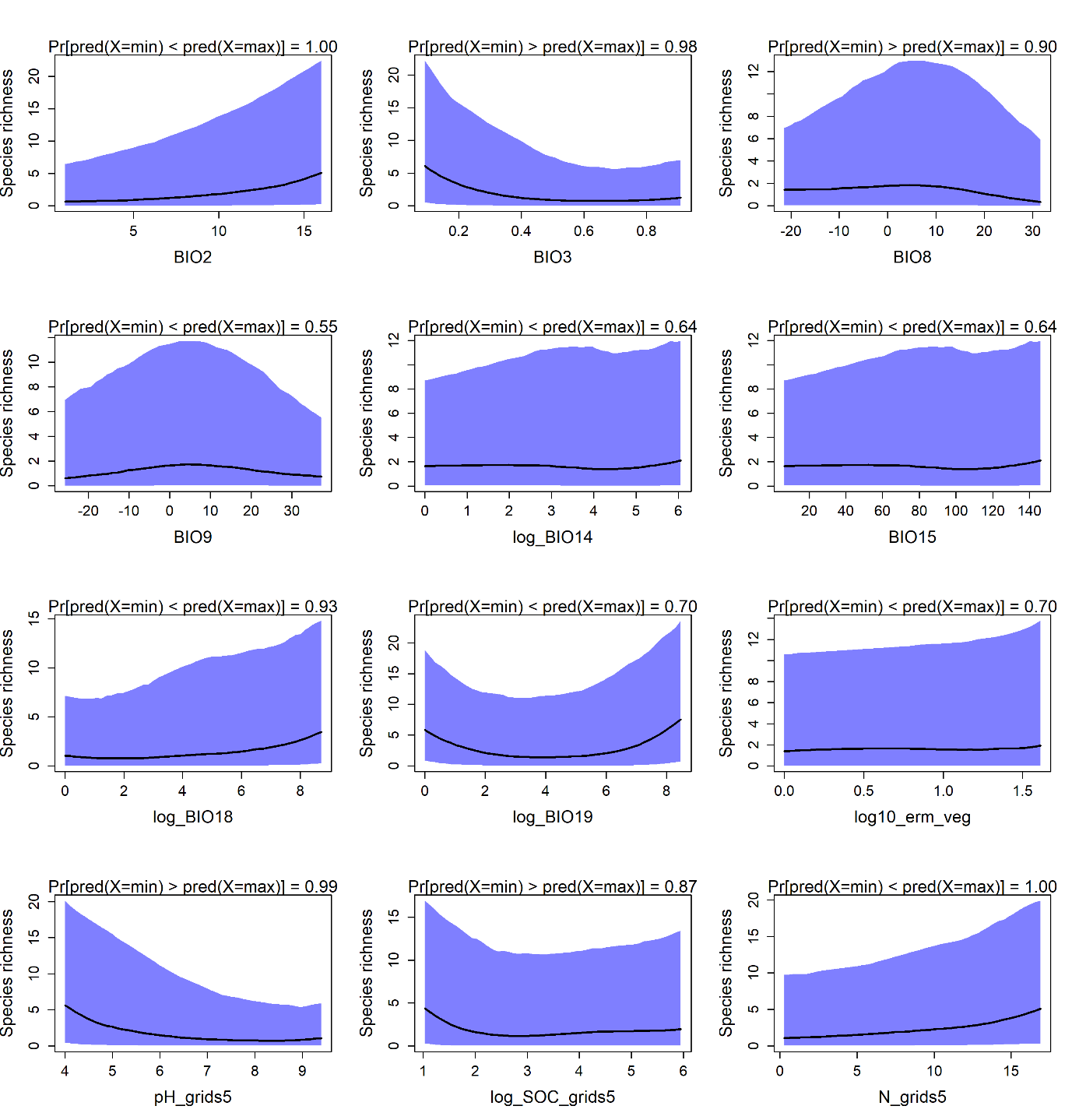


**Fig. S8.** Marginal associations (i.e., associations with a given variable after accounting for the effects of all other variables) of explanatory variables with ErM fungal species richness, computed from the Hmsc model. BIO2: mean diurnal air temperature range; BIO3: isothermality; BIO8: mean daily air temperatures of the wettest quarter; BIO9: mean daily air temperatures of the driest quarter; BIO14: precipitation amount of the driest month; BIO15: precipitation seasonality; BIO18: mean monthly precipitation amount of the warmest quarter; BIO19: mean monthly precipitation amount of the coldest quarter.


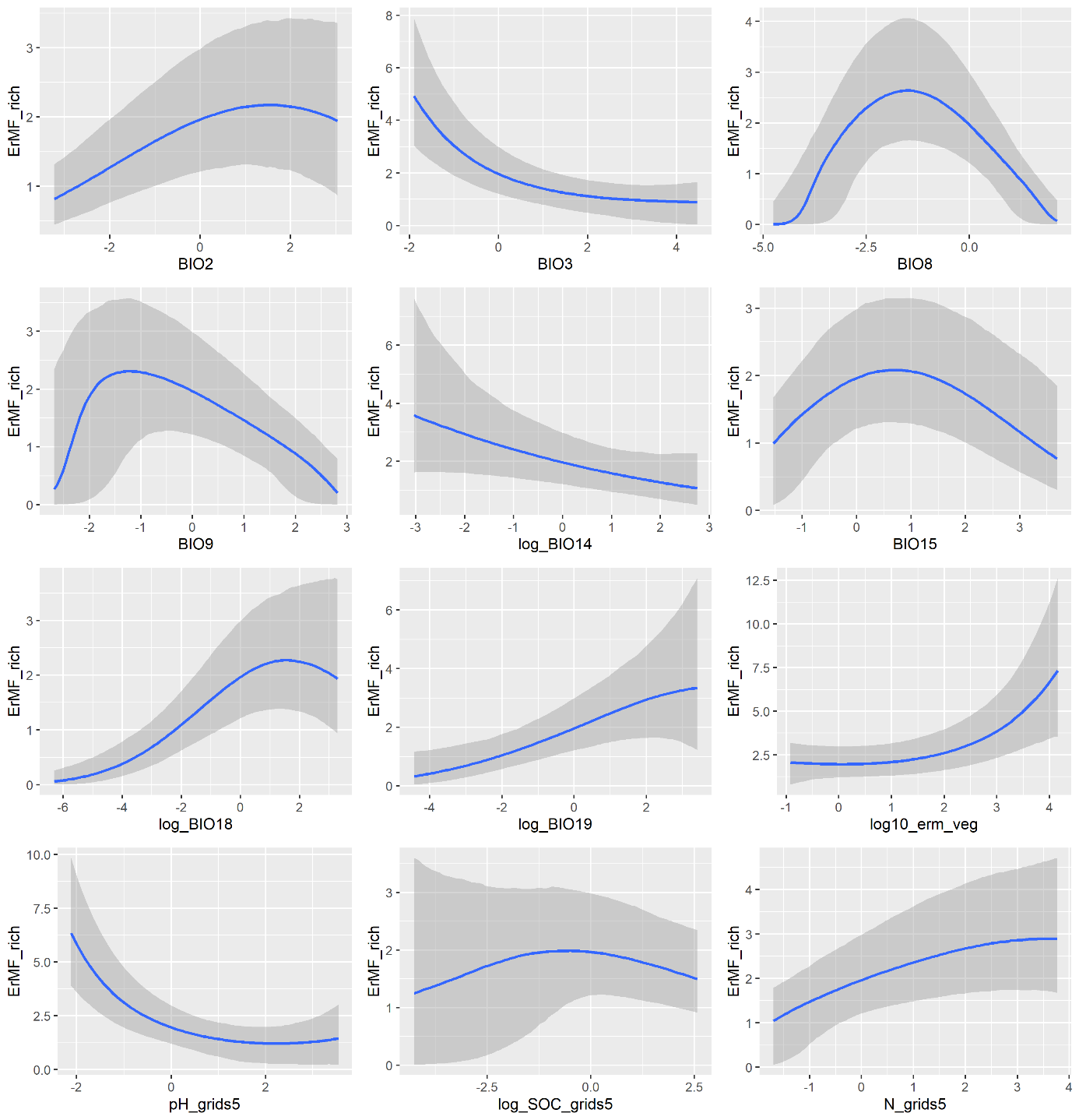


**Fig. S9**. Marginal associations (i.e., associations with a given variable after accounting for the effects of all other variables) of explanatory variables with ErM fungal species richness, computed from the ZIP model. BIO2: mean diurnal air temperature range; BIO3: isothermality; BIO8: mean daily air temperatures of the wettest quarter; BIO9: mean daily air temperatures of the driest quarter; BIO14: precipitation amount of the driest month; BIO15: precipitation seasonality; BIO18: mean monthly precipitation amount of the warmest quarter; BIO19: mean monthly precipitation amount of the coldest quarter.


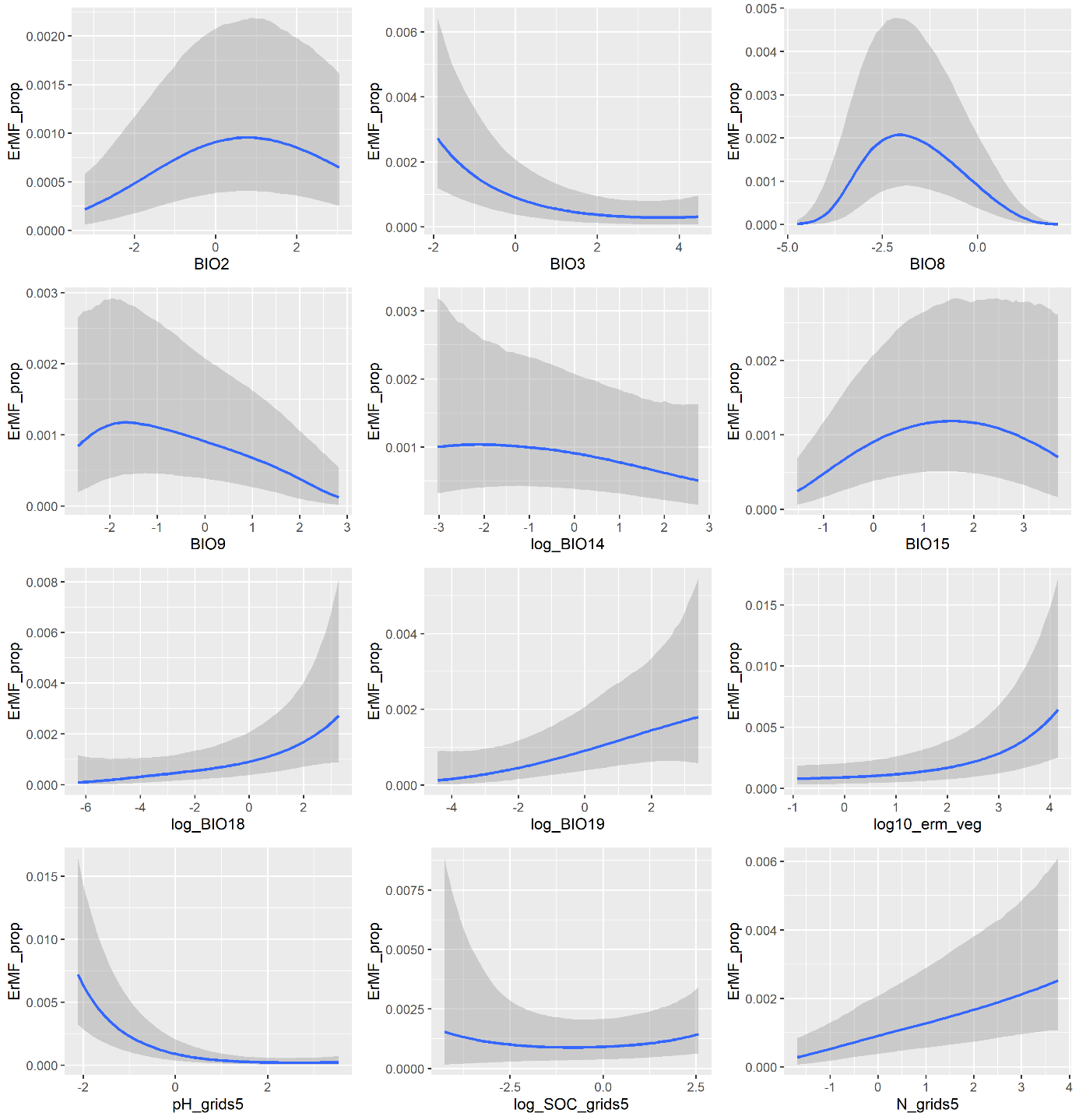


**Fig. S10**. Marginal associations (i.e., associations with a given variable after accounting for the effects of all other variables) of explanatory variables with ErM fungal relative abundance, computed from the ZIB model. BIO2: mean diurnal air temperature range; BIO3: isothermality; BIO8: mean daily air temperatures of the wettest quarter; BIO9: mean daily air temperatures of the driest quarter; BIO14: precipitation amount of the driest month; BIO15: precipitation seasonality; BIO18: mean monthly precipitation amount of the warmest quarter; BIO19: mean monthly precipitation amount of the coldest quarter.


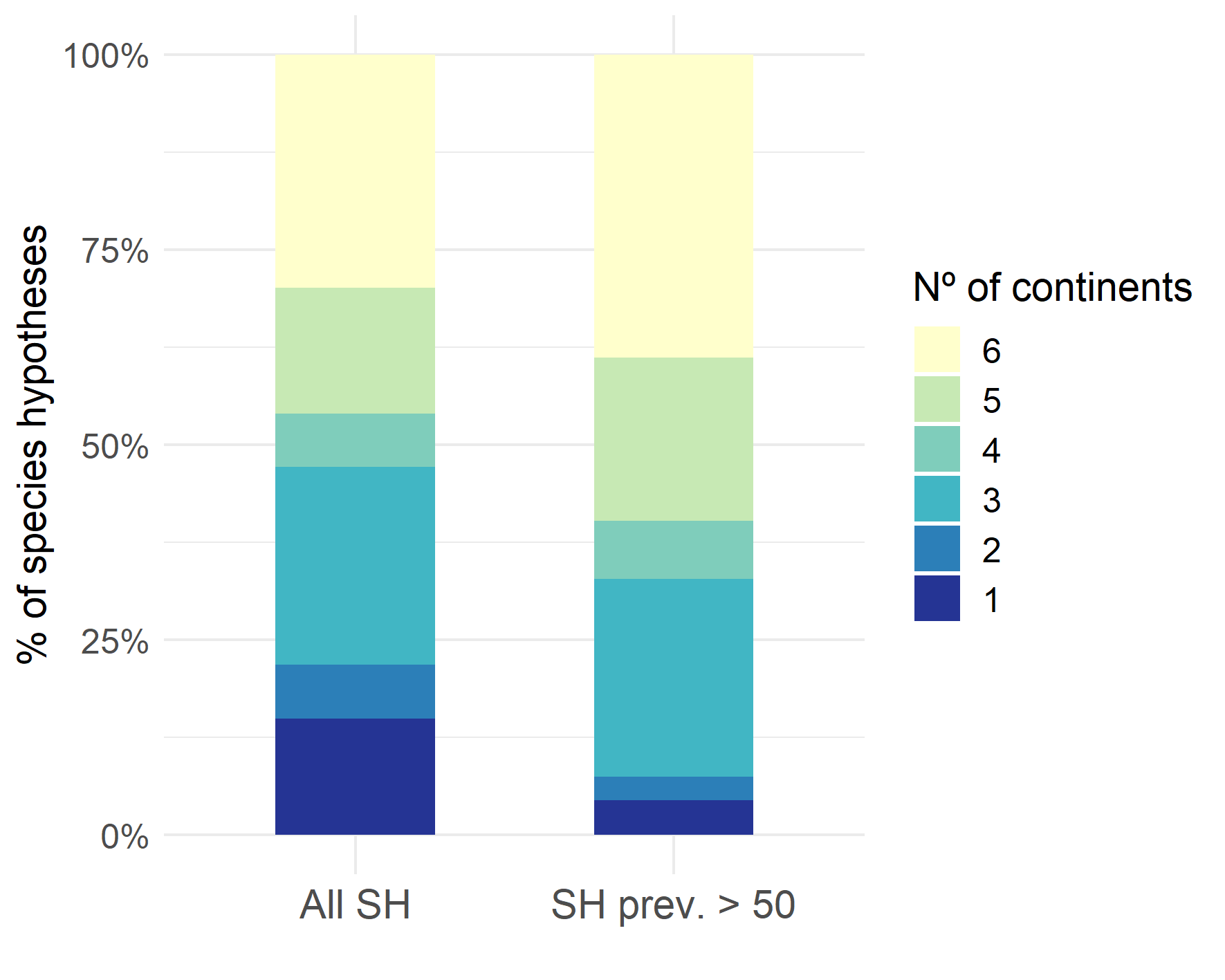


**Fig. S11.** Distribution of number of the occurrences of species hypotheses in different numbers of continents. First stacked bar includes all observed species hypotheses, whereas the second includes the species hypotheses observed in more than 50 samples in the dataset.


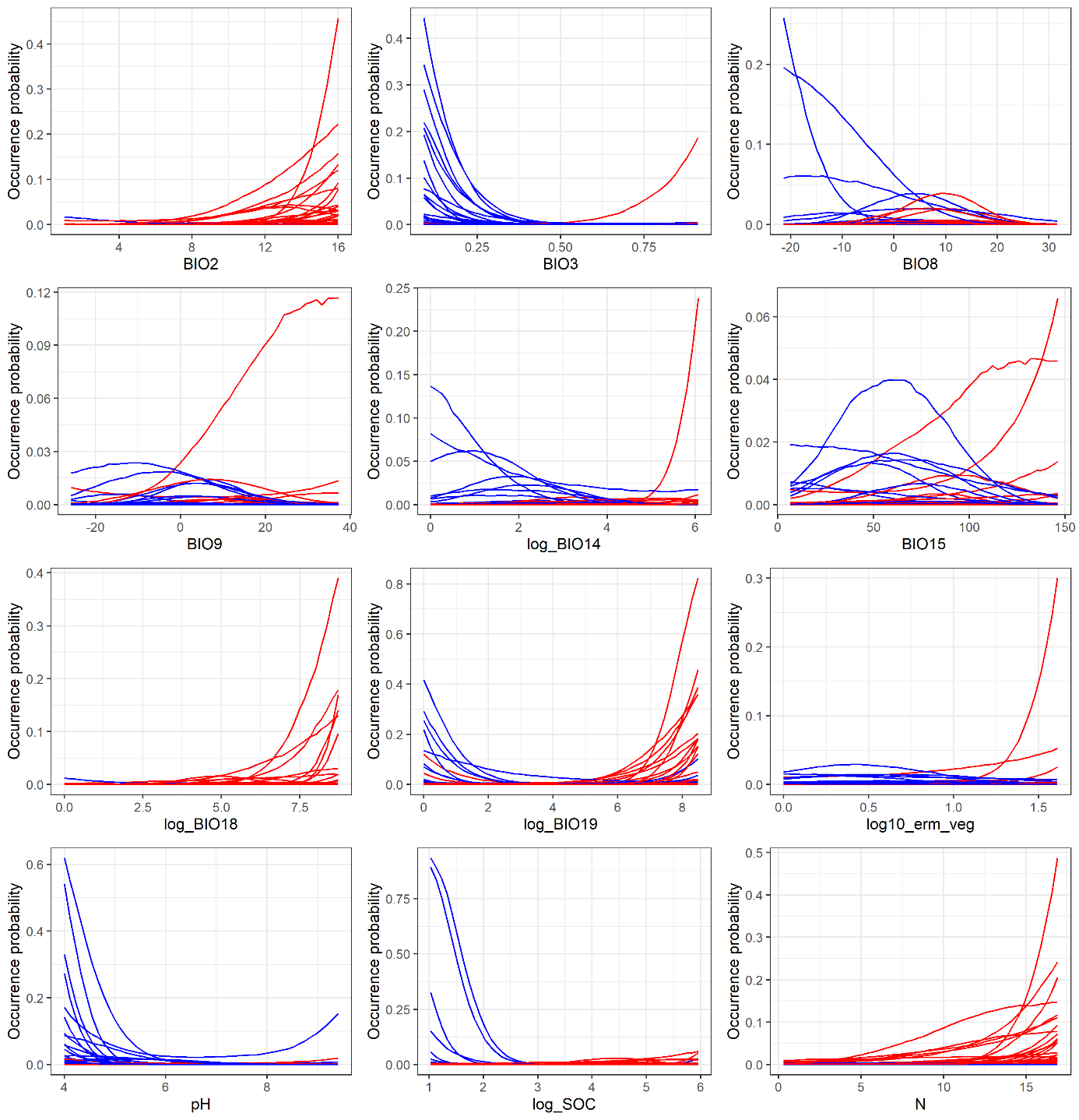


**Fig. S12.** Marginal associations of explanatory variables with ErM fungal species occurrences, computed from the Hmsc model. Decreasing trends are depicted in blue and increasing trends are depicted in red. BIO2: mean diurnal air temperature range; BIO3: isothermality; BIO8: mean daily air temperatures of the wettest quarter; BIO9: mean daily air temperatures of the driest quarter; BIO14: precipitation amount of the driest month; BIO15: precipitation seasonality; BIO18: mean monthly precipitation amount of the warmest quarter; BIO19: mean monthly precipitation amount of the coldest quarter.


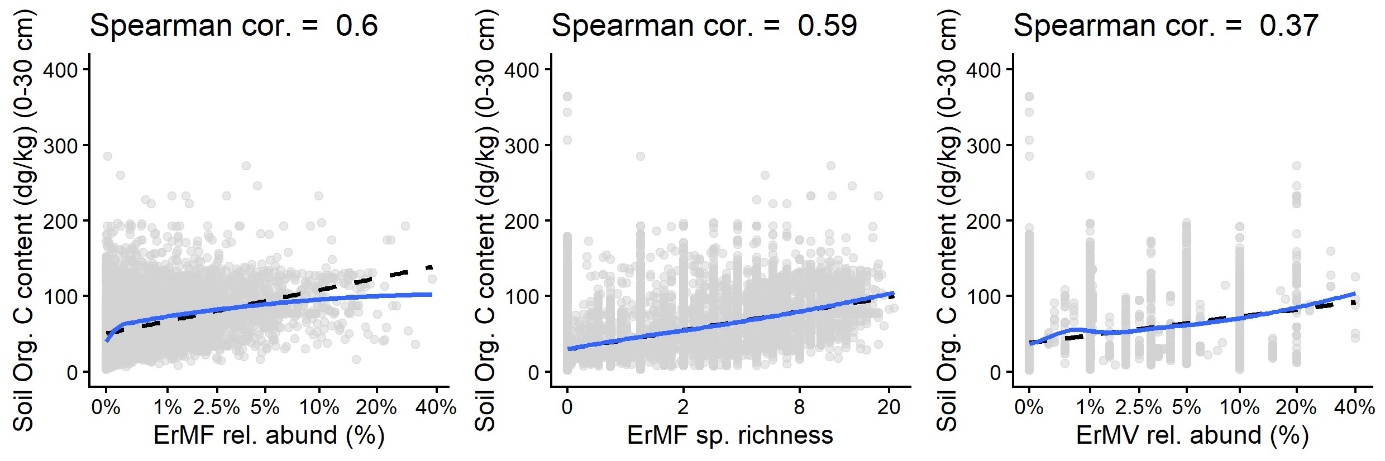


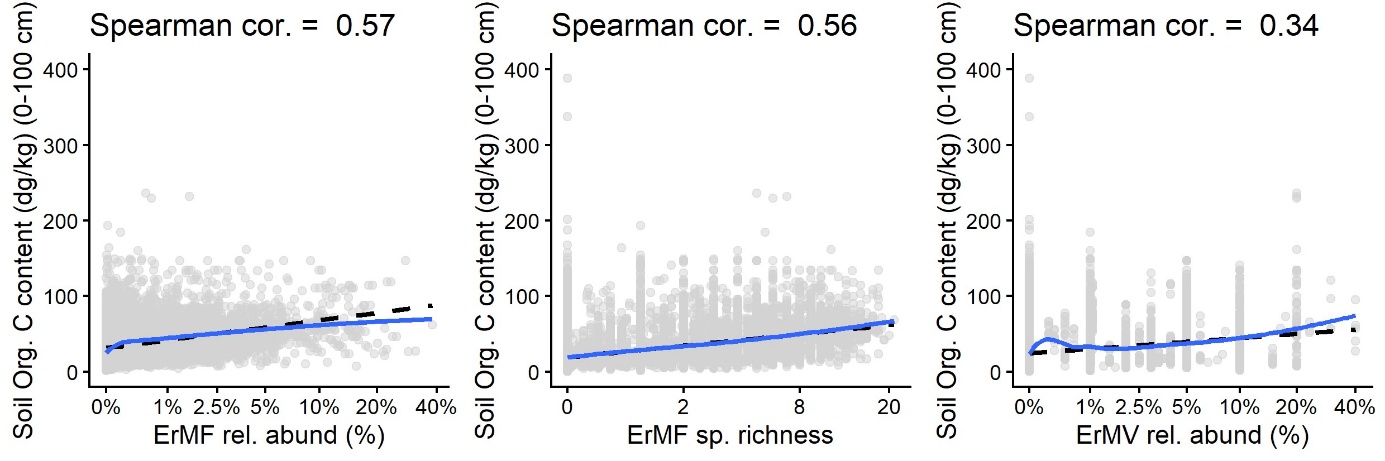


**Fig. S13.** Correlations between global soil organic carbon content at depths 0-30 cm (upper panels) and 0-100 cm (bottom panels), and Ericoid mycorrhizal fungal relative abundance (left panels), Ericoid mycorrhizal fungal species richness (middle panels) and Ericoid mycorrhizal vegetation relative abundance (right panels). Dashed black line captures the linear relationship whereas the blue smooth line corresponds to a LOESS fit. Note the log scale of the x-axes.


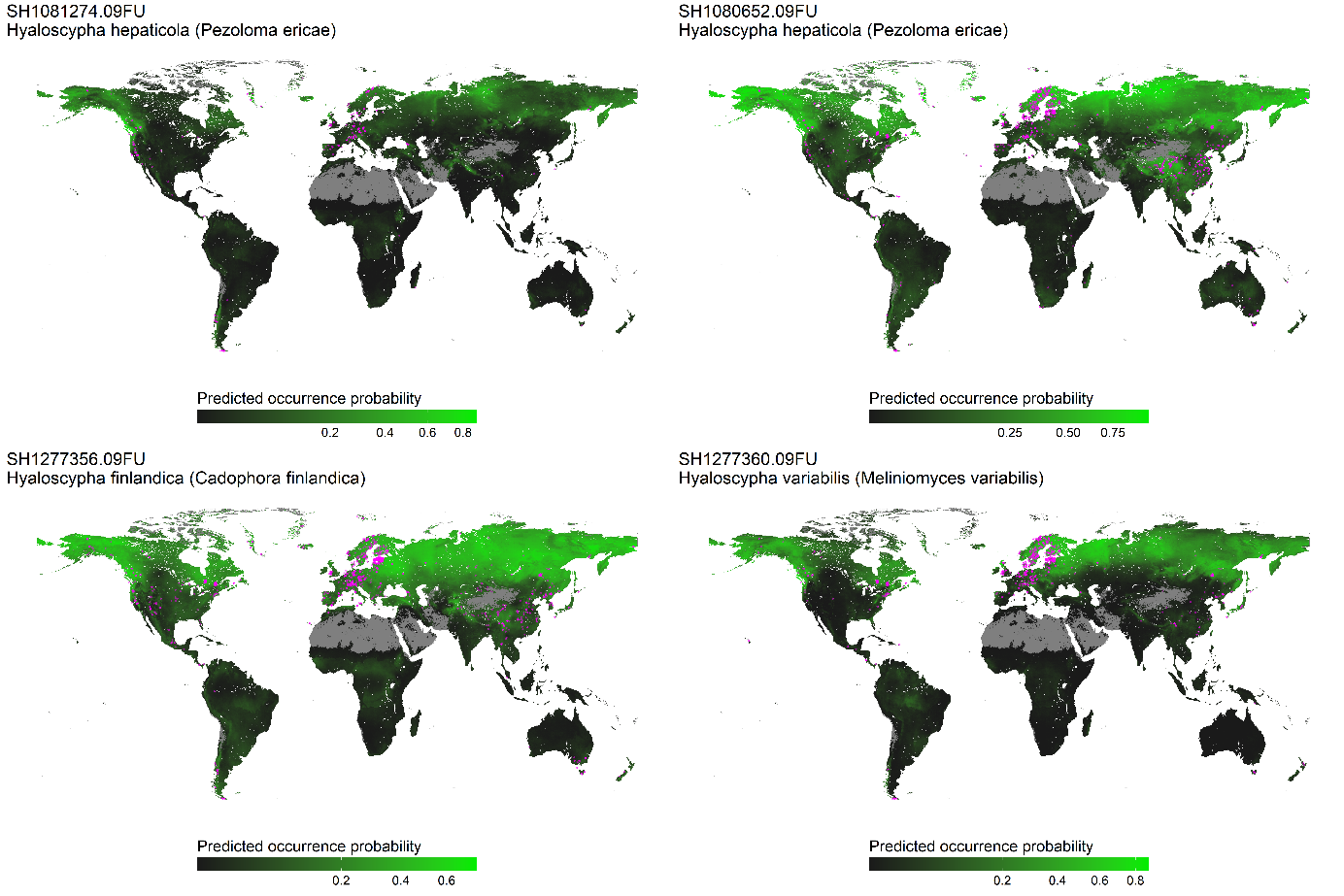


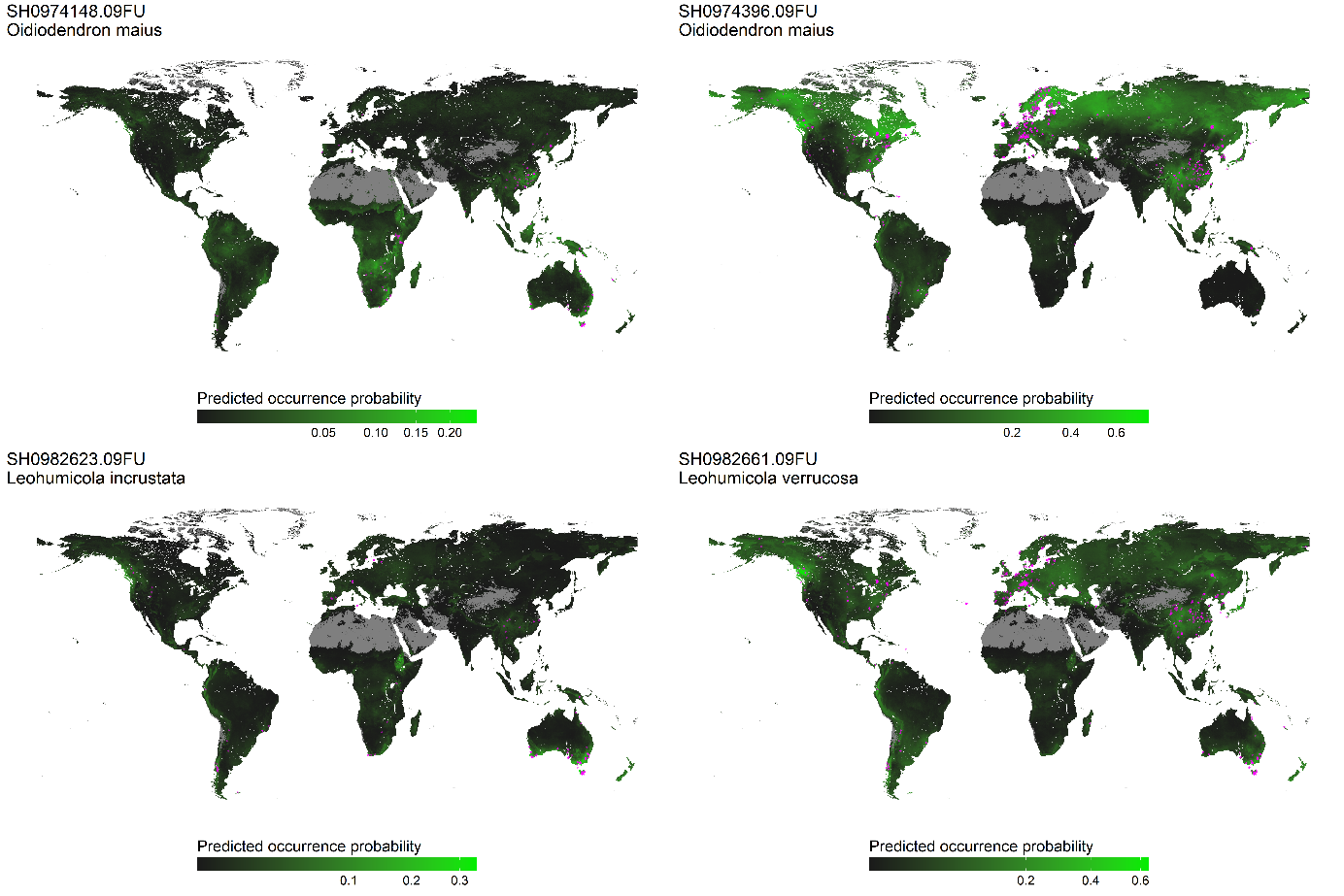


**
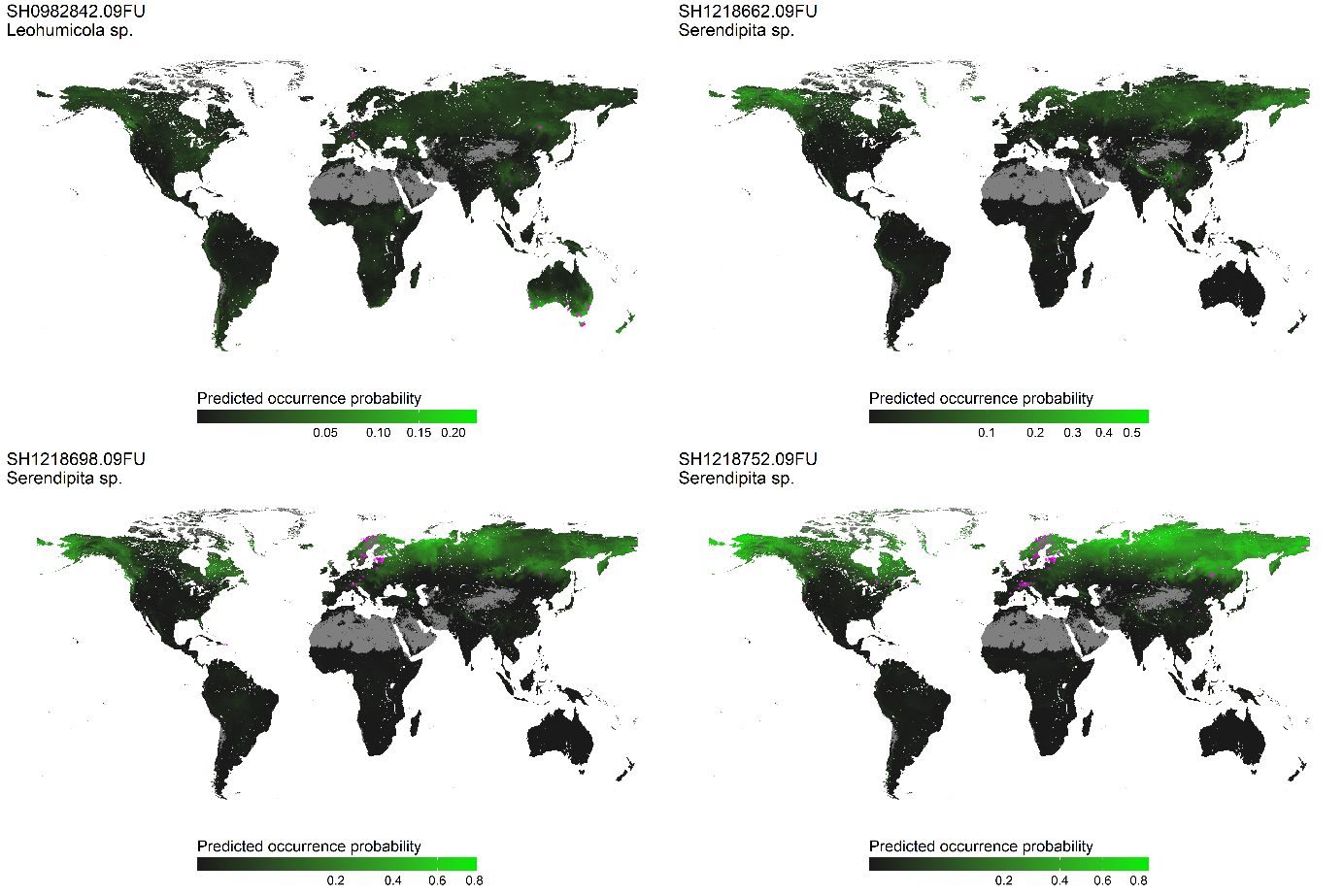
**

**Fig. S14.** Global distribution maps of common ErM fungal taxa. Magenta dots indicate the locations where the taxa have been actually recorded. Masked locations (grey) show sparsely vegetated zones and dense urban areas based on global land-cover data. Note the square root color scale.
